# DeepRepeat: direct quantification of short tandem repeats on signal data from nanopore sequencing

**DOI:** 10.1186/s13059-022-02670-6

**Published:** 2022-04-28

**Authors:** Li Fang, Qian Liu, Alex Mas Monteys, Pedro Gonzalez-Alegre, Beverly L. Davidson, Kai Wang

**Affiliations:** 1 Raymond G. Perelman Center for Cellular and Molecular Therapeutics, Children’s Hospital of Philadelphia, Philadelphia, PA 19104 USA; 2 School of Life Sciences, College of Science, University of Nevada, Las Vegas, 4505 S Maryland Pkwy, Las Vegas, NV 89154 USA; 3 Nevada Institute of Personalized Medicine, College of Science, University of Nevada, Las Vegas, 4505 S Maryland Pkwy, Las Vegas, NV 89154 USA; 4 Department of Pathology and Laboratory Medicine, Perelman School of Medicine, University of Pennsylvania, Philadelphia, PA 19104 USA

**Keywords:** Short tandem repeat, Nanopore sequencing, Deep learning, Telomeric repeat

## Abstract

Despite recent improvements in basecalling accuracy, nanopore sequencing still has higher error rates on short-tandem repeats (STRs). Instead of using basecalled reads, we developed DeepRepeat which converts ionic current signals into red-green-blue channels, thus transforming the repeat detection problem into an image recognition problem. DeepRepeat identifies and accurately quantifies telomeric repeats in the CHM13 cell line and achieves higher accuracy in quantifying repeats in long STRs than competing methods. We also evaluate DeepRepeat on genome-wide or candidate region datasets from seven different sources. In summary, DeepRepeat enables accurate quantification of long STRs and complements existing methods relying on basecalled reads.

**Supplementary Information:**

The online version contains supplementary material available at 10.1186/s13059-022-02670-6.

## Background

Short tandem repeats (STRs), also known as microsatellites, are directly adjacent repetitions of specific nucleotide motifs in a genome. STR units are diverse with various compositions of nucleotides and of different repeat unit sizes (generally ranging from 1 to 10). For example, GGGGCC is a hexanucleotide STR motif in C9orf72 that is associated with amyotrophic lateral sclerosis (ALS), while CAG is a well-known trinucleotide repeat motif in several genes associated with repeat expansion disorders. More than half million STRs have been catalogued from the reference genome, covering approximately 3% of the human genome [1]. Repeat expansions of specific STRs, including those in exons, UTRs or introns, are known to cause various human diseases. To date, over 40 neurological disorders have been found to be associated with trinucleotide repeat expansion, including Huntington’s diseases [2], the spinocerebellar ataxias [3], fragile X syndrome [4], Friedreich’s ataxia [5], and others [6–8]. Additional human diseases caused by repeat expansions have been found recently [9–19], partly because of the recent development of long-read sequencing technologies. In various repeat expansion diseases, repeat count of STR units is found to correlate inversely with the age of onset: larger repeat expansions are usually associated with earlier age onset of the disease [20, 21]. Thus, the identification of novel STRs and the quantification of repeat counts in known STRs are critically important to the study of repeat-associated human diseases and the eventual development of therapeutic strategies.

The current approaches to determining repeat counts of STRs include Southern blot analysis, capillary electrophoresis, and, in some cases, Sanger sequencing. However, these techniques are labor intensive and time-consuming for large-scale STR detection and cannot be applied at genome-wide scale. Next generation sequencing, such as Illumina short-read techniques, paved a way for genome-wide STR detection, and various computational methods have been developed for assisting this task, such as lobSTR [22], RepeatSeq [23], STRviper [24], TREDPARSE [25], HipSTR [26], ExpansionHunter [27], and STRetch [28]. However, the length of reads in short-read techniques usually ranges from 100 to 150 bp (base pairs), which are much smaller than pathogenic STR expansions that are hundreds of or even thousands of base pairs in length. Thus, short-read techniques have limited power to detect pathogenic STR expansions. Long-read sequencing techniques, such as PacBio and Oxford Nanopore sequencing, can generate much longer reads with up to hundreds of thousands of bp. Longer reads can provide better alignment for pathogenic STRs using information from flanking sequences, and several computational tools have been developed to detect repeat counts based on long reads, including RepeatHMM [29], Tandem-genotypes [30], RepLong [31], and TRiCoLOR [32]. These tools together with long-read techniques have achieved great success in characterizing disease-associated STRs [9–12]. One drawback to long read data is the relatively high basecalling error rate (3 to 15%) [33] (although different strategies, such as linear consensus [34] or amplicon-based UMIs [35], were designed to reduce errors, additional cost is needed and the scalability is limited). More importantly, one less known limitation is that basecalling errors of nanopore sequencing are not uniformly distributed across genome in long-read data; instead, they are becoming much higher in repetitive regions (see the analysis below) when the size of repeat regions is very large (which is the case when there are repeat expansions in patient samples). Therefore, while existing approaches can use basecalled reads to analyze short STRs in healthy subjects, there can be substantial challenges in characterizing disease-associated and highly expanded STRs that are specifically observed in patients.

Signal data has been used to improve STR detections and reduce the bias in STR regions caused by higher error rates, before recent improvements in basecalling accuracy is made. De Roeck et al. proposed NanoSatellite to detect STRs at the nanopore squiggle level using dynamic time warping and detected a 25 bp repeat unit, where the expanded alleles strongly increase risk of Alzheimer’s disease [36]. Meanwhile, Giebelmann et al. used profile hidden Markov model to identify STR regions and upstream/downstream flanking sequences with the help of signal alignment to flanking regions, and their tool STRique has been evaluated on GGGGCC repeats of FTD/ALS (Frontotemporal Dementia and Amyotrophic Lateral Sclerosis) synthetic sequences [37]. However, these tools rely on synthetic signals for various STR units and their flanking regions, which deviate from real signals. As evidenced in existing works [38–40], real signals are commonly observed as a distribution rather than a signal value which is used as synthetic signals for a specific pore model. Therefore, analysis on datasets encountered in real-world settings is important to understand the characteristics of different computational approaches.

In this study, we propose a deep learning tool, DeepRepeat, to accurately detect STRs directly from nanopore electric signals, without using synthetic signals. DeepRepeat is based on the notion that directly adjacent STR units share similar nanopore signal distribution. It then converts real signals of a STR unit and its upstream and downstream STR units into RGB channels of a color image, where the height represents the signal range and the width represents STR unit size. Subsequently, we feed repeat and non-repeat images into a deep convolutional neural network followed by a full connection network for classification. Based on alignment of all long reads for a specific STR locus in an individual, the information is summed from multiple long reads for the STR locus using a Gaussian mixture distribution. We evaluate DeepRepeat for repeat identification and count estimation on multiple STR datasets. Of particular interest in our evaluation is the CHM13 genome, which is the first telomere-to-telomere genome assembly that was recently published using a collection of sequencing approaches through consortium efforts. It is an interesting testing case because of the ability to resolve telomeric repeats accurately and because of the accurate quantification of long STR regions (unlikely to be pathogenic though). We also evaluated DeepRepeat on multiple datasets on healthy subjects and those with known repeat expansions. Our experiments show several unique advantages of DeepRepeat over existing methods. DeepRepeat is publicly available at https://github.com/WGLab/DeepRepeat .

## Results

### Overview of DeepRepeat

The input of DeepRepeat is basecalled nanopore data with electric (ionic) signals, and the output is estimated repeat counts (Additional file 1: Fig. S1). There are three steps in DeepRepeat: converting signals to images, using deep learning for STR prediction on each nucleotide, and summarizing the predictions on multiple reads to infer repeat counts.

**Fig. 1:**
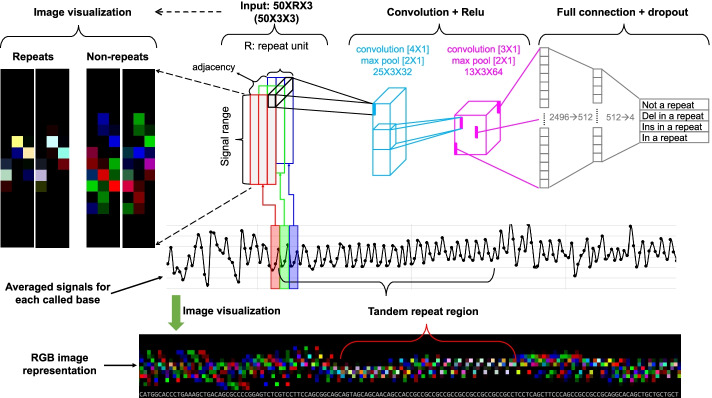
DeepRepeat workflow: converting nanopore signals to images for deep learning prediction. Each dot in the curve in black represents an event in nanopore data and is represented by a column in a channel. R: the size of repeat motif, for example, R = 3 for trinucleotide repeats. Del: deletion indicating 1 bp deletion compared against the repeat motif of interest; Ins: insertion indicating 1 bp insertion compared against the repeat motif of interest. In the bottom and the left, an ideal image representation of repeat regions is white dots, while the image representation of non-repeat regions is dispersed red or blue or green or their mixture. Each basecalled nucleotide in the sequence of the bottom subfigure is for a column for demonstration purpose

One novel and key step of DeepRepeat is to convert electric signals to images, which is illustrated in Fig. 1 with trinucleotide repeat as an example for simplicity. In DeepRepeat, signal distribution of each STR unit is transformed into a matrix, and three matrices from the three consecutive STR units are converted as three channels of a color image. Then, the self-similarity between adjacent repeat copies in a STR region is used to infer whether nucleotides are in a specific STR region. With this property in mind, an image of three consecutive STR units is expected to have signals at the same or close corresponding positions in each channel with a color close to white, and the rest of image is black. In an ideal case, images of repeats only have several white dots with black as background, while images of non-repeats have many mixed colors that are randomly dispersed. Using this transformation, the STR detection is creatively converted to an image recognition problem, and then deep convolutional network in the field of image processing is used to identify STR units from nanopore signals. Finally, after making the prediction of whether a base in long reads is in a repeat region or not, the long reads are aligned against a reference genome, and the results on multiple reads are summarized to quantify the repeat counts using a Gaussian mixture model.

In this section below, we will first demonstrate higher error profiles of repeat expansion regions, which is a little-known fact because most researchers do not focus analysis on highly expanded repeat region. We then evaluate DeepRepeat on individual loci and on a genome-wide scale, including assessment on (i) telomeric/long STR regions in CHM13, (ii) a CAG repeat dataset of the *HTT* gene of 11 Huntington’s disease samples, (iii) a GGGGCC repeat dataset of synthetic sequences [37], (iv) nine STR loci with validation by Sanger sequencing, (v) 57 manually curated STR loci for two different genomes (HX1 and NA12878), and (vi) genome-wide prediction of STR bases in two genomes (HX1 and NA12878) with cross-genome independent testing strategy.

### Estimation of mismatch error of repeat regions

We used two published nanopore long-read datasets [37] to estimate how repeat expansion affects basecalling errors in Oxford Nanopore sequencing. We performed re-basecalling using two recently released algorithms, including Guppy v3.3.3 and v5.0 (the two Guppy versions differ substantially in the underlying algorithms) together with Albacore. One dataset represents synthetic sequences with five distinct repeat counts (8, 32, 50, 56, and 76) of the GGGGCC motif, while the repeat locus of the reference sequence has 10 repeat counts. These long reads from five groups have highly similar flanking regions of repeat but different repeat counts, and a general alignment process will complicate further analysis. Thus, we generated four different reference sequences with 8, 32, 53, and 76 repeat counts (repeat counts 50 and 56 are close to each other, and thus are considered together), and then aligned all long reads to the reference sequences using minimap2 [41]. Note that a long read may be aligned against multiple reference genomes, but only one of them is considered as primary alignment with the maximum alignment score, and the primary alignments should be aligned against the reference sequence with the closest benchmark repeat counts. For example, if a long read has a primary alignment against a reference with 32 repeat counts, it is considered to be from a group with 32 repeat counts. With this strategy, all long reads were assigned into different groups.

**Fig. 2:**
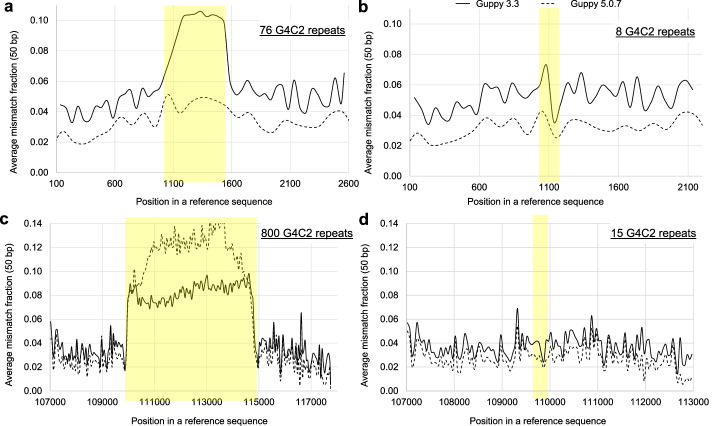
Mismatch error rate for nanopore long reads with different repeat lengths. Repeat regions are highlighted in yellow. **a** and **b** have 76 and 8 G4C2 repeat copies for 2 synthetic sequences respectively, while **c** and **d** have ~ 800 and 15 repeat copies for a BAC clone. Each dot is for an averaged mismatch error of a 50 bp region to reduce randomness. The long-read data were released by [37] and rebasecalled via Guppy v3.3.3 (solid lines) and Guppy 5.0.7 (dashed lines). A summary of error rate statistics is shown in Additional file 3

We then analyzed the mismatch error for the repeat regions and the flanking regions (Fig. 2). We found that for the reads belonging to the group with 8 repeats, the mismatch error rate (5.6%) in the repeat region is higher than that (3.6%) in the flanking region (Fig. 2b). However, when the repeat size expands to 76 (Additional file 1: Fig. S2), the mismatch error rate in the repeat region increases to 10%, which is 78% higher than that of the flanking region (5.6%) (Fig. 2a). The above analysis was based on Guppy v3.3.3 basecalling. We also conducted a similar analysis on nanopore reads basecalled via Albacore v2.3, and the conclusion is similar (Additional file 1: Fig. S4). All the analysis indicates that the mismatch error rate significantly increases as repeat size is becoming larger. Basecalling errors by Guppy 5.0.7 decrease, but are still higher in much longer repeat regions as shown below.

We analyzed another dataset of a BAC clone [37] where the repeat copies could be up to 800 GGGGCC units. Using a similar strategy above, we investigated the mismatch error of repeat regions. The results shown clearly demonstrate that the repeat region with 800 copies has much higher mismatch error rate than its flanking regions (Fig. 2c, d, Additional file 1: Fig. S3). In contrast, the repeat region with 15 copies has similar mismatch error rate with the flanking regions (Fig. 2d). Similarly, Giesselmann et al. also concluded that single-read base calling at the genomic level becomes unreliable in long G4C2 repeats [37]. These results demonstrate that the mismatch error rate of nanopore sequencing can increase substantially in genomic regions with highly expanded STRs, which may bias results from algorithms that rely on basecalled reads rather than raw nanopore signals.

### Detection of long STRs in CHM13

We next tested DeepRepeat on detection of long repeats in the CHM13 genome, using models trained on NA12878 and HX1. CHM13 is a well-studied genome with both PacBio HiFi and Oxford Nanopore long-read data publicly available [42]. The CHM13 cell line has near-complete homozygosity, with a few exceptions in specific regions. The telomere-to-telomere consortium has finished the de novo assembly of CHM13 primarily based on HiFi reads, but supplemented with data from other short- and long-read platforms. Since CHM13 assembly is shown to be highly reliable, repeat counts from the assembly can be considered as a benchmark to evaluate the detection of long repeats from nanopore data only. In this subsection, the benchmark “true” repeat counts in CHM13 are directly calculated from the published assembly (v1.1).

**Fig. 3:**
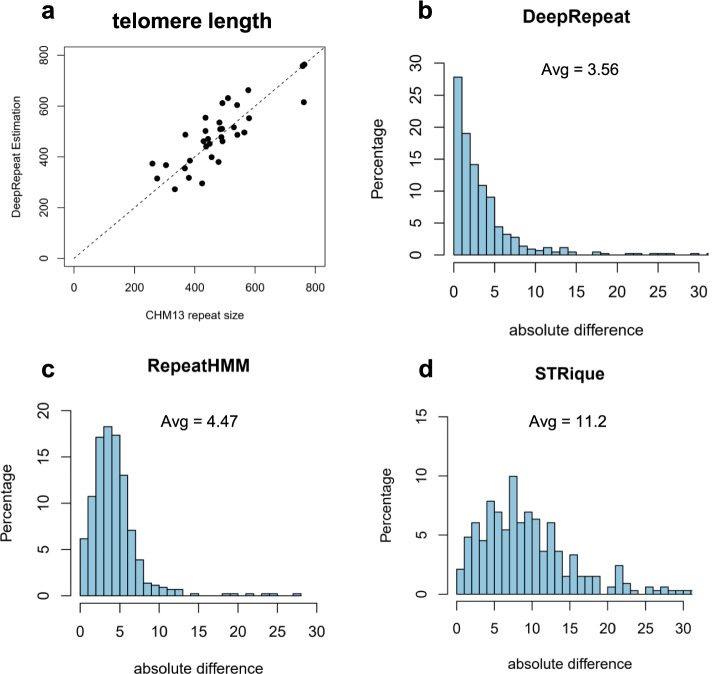
Benchmarking repeat quantification on CHM13. **a** Estimated telomeric repeat counts against benchmark repeat counts determined on CHM13 v1.1 assembly (Pearson correlation = 0.83). **b**–**d** Quantification of 439 long STR regions in CHM13 using DeepRepeat (**b**), RepeatHMM (**c**), and STRique (**d**). The distribution of absolute difference between estimated repeat counts and the truth set (CHM13 v1.1 assembly) is shown

We first tested DeepRepeat on the detection of telomeric repeats in CHM13. Each telomere was quantified separately using 30X nanopore data of CHM13. Quantification of telomere repeat is challenging because the repeat is at the end of the chromosome and only one flanking region is available. After removing reads that are not confidently mapped to the flanking region, the average number of reads per telomere was 20. The comparison of DeepRepeat estimation and CHM13 assembly is shown in Fig. 3. The estimation of DeepRepeat is consistent with the CHM13 assembly (Pearson correlation = 0.83), but with some variations. The reasons might be that (1) we did not use the raw signals of the full data set (126X) due to storage limitations; (2) the repeat count variation in the telomere regions is larger than regular STR regions because the telomere length can be mosaic in a cell line; and (3) since there is only one flanking region for a given telomere, it is unknown if the full length of the telomere had been sequenced completely for each read. The standard deviations of repeat count between reads are about 100 in the telomere regions. Two other tools, RepeatHMM and STRique [37] cannot detect this type of repeat (either detected nothing or reported an error). This is not surprising because other repeat estimation methods need accurate alignment of reads against both flanking sequences of repeat regions. To the best of our knowledge, DeepRepeat is the only tool that quantifies telomeric repeats and generates an overall estimation as well as a per-read estimation using signal data.

Next, we benchmarked DeepRepeat, RepeatHMM [29], and STRique [37] (a method using synthetic signals) on 439 STR regions on the CHM13 genome. These are all STR regions that are > 200 bp and not within a 500-bp flanking region of another STR. We removed adjacent STRs because many of the adjacent STRs have similar sequences and it is hard to tell if they need to be merged or not without manual examination. The lengths of the 439 STR regions range from 200 bp to 2374 bp, and their coordinates are shown in Additional file 2: Table S1. The evaluation results are shown in Fig. 3, where it is clear that DeepRepeat achieves better performance than RepeatHMM and STRique. For example, the average absolute difference between estimated and benchmark repeat counts is 3.56, 4.47, and 11.2 for DeepRepeat, RepeatHMM and STRique, respectively. DeepRepeat reduces the estimation errors by 20% compared to RepeatHMM and 68% compared to STRique. Thus, DeepRepeat outperforms existing methods to detect very long STR repeats.

### Detection of CAG repeats in *HTT* on Huntington’s disease cell lines

**Fig. 4:**
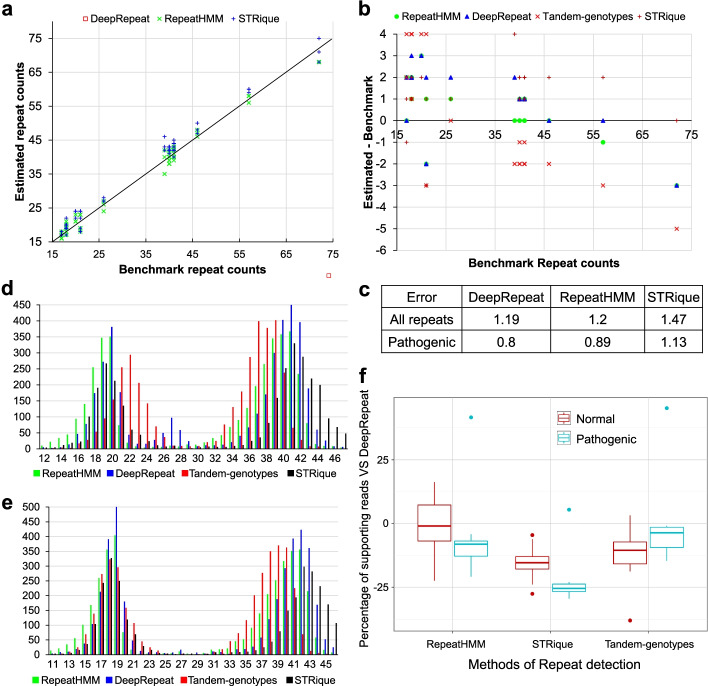
Repeat count estimation on 11 Huntington’s disease samples with CAG repeats and on NA12878. **a** The correlation of estimated repeat counts against benchmark repeat counts on ~15X downsampling data (3 times for each data). **b** The difference of estimated repeat counts minus benchmark repeat counts for the four methods. **c** Averaged absolute difference (Error) of estimated repeat counts and benchmark repeat counts. “All” for all repeat counts, while “pathogenic” for only pathogenic repeat counts. **d** The distribution of estimated repeat counts from all long reads for a sample (ND30422) whose benchmark repeat counts are 18 and 40 (shown by a down arrow in magenta). **e** The distribution of estimated repeat counts from all long reads for another sample (ND30626) whose benchmark repeat counts are 21 and 41 (shown by a down arrow in magenta). The distribution for more samples is in Additional file 1: Fig. S5. **d**, **e: ***x*-axis for estimated repeat counts; *y*-axis for the number of supporting reads for each estimated repeat count. **f** The difference of supporting reads between DeepRepeat and other methods (in percentage = (o-*D*)*100/*D* where *D* is the number of supporting reads by DeepRepeat, while “o” is the number of supporting reads by other methods (RepeatHMM, Tandem-genotypes and STRique)

We next tested DeepRepeat for the estimation of repeat counts for 11 Huntington’s disease (HD) cell lines, using the model trained on an in-house nanopore data set of HD samples. HD is a neural degenerative disease caused by CAG repeat expansions in exon-1 of the *HTT* gene. In this study, we generated high coverage (~4000X) targeted nanopore sequencing data of the *HTT* exon-1 region of 11 HD cell lines and also performed Sanger sequencing to determine the repeat counts. The repeat counts of the pathogenic expanded allele for the 11 samples range from 39 to 72, while the repeat counts for normal allele are between 17 and 21 with one exception of 26. We run DeepRepeat, RepeatHMM [29], Tandem-genotypes [30], and STRique [37] on this data, and compared their performance against repeat counts inferred from Sanger sequencing. The results are shown in Fig. 4.

First, we downsampled the high coverage nanopore sequencing data to 15X coverage to mimic whole-genome sequencing data with lower coverage (i.e. a PromethION cell on a single human genome) and tested the accuracy of DeepRepeat, RepeatHMM and STRique. This process was repeated 3 times, and the results are shown in Fig. 4a, c. All the three methods have very high Pearson’s correlation with the benchmark (> 0.99), but DeepRepeat performs better than the others. Meanwhile, we also calculated averaged absolute error of each method. The averaged absolute errors (as shown in Fig. 4c) are 1.19, 1.2, and 1.47 for DeepRepeat, RepeatHMM and STRique, respectively. We also calculated the average absolute errors for pathogenic alleles only, because pathogenic repeats are more important for clinical diagnostic purposes. The error rates are 0.8, 0.89, and 1.13 for DeepRepeat, RepeatHMM and STRique, respectively. In both cases, DeepRepeat achieves lower error than other methods. Of note, DeepRepeat achieves much lower error and is several times faster than STRique (both of them analyze nanopore signals data; speed comparison is described in the section below).

We also tested the methods on the original high-coverage data, and the results are shown in Fig. 4b. All four methods provide accurate estimation of repeat counts with the difference of estimated repeat counts and benchmark repeat counts between − 5 and 4, and estimated repeat counts have excellent correlations with benchmark repeat counts. In practice, this high-coverage dataset is far more than enough for DeepRepeat. We generated this because it is a targeted sequencing and the MinION flow cell has more capacity than needed.

To further examine how long reads support estimated repeat counts for a sample, we illustrated the distribution of estimated repeat counts of long reads in Fig. 4d and e for two samples (the distribution for other samples is shown in Additional file 1: Fig. S5). All four methods show clear peaks around the true repeat counts. However, DeepRepeat has more supporting reads to infer repeat counts, especially for the pathogenic (expanded) allele (supporting reads for a called allele are those reads with similar repeat counts to the peak of each method. Same thresholds were used for different methods). This is particularly important, since typically fewer reads are available for longer alleles vs. normal alleles due to differences in sequencing efficiency and amplicon generation. To evaluate how many more long reads with expected repeat counts can be used for STR detection, we summarized the number of supporting reads for inferring repeat counts in Fig. 4f and Additional file 1: Table S2. For pathogenic STR alleles, DeepRepeat detected ~ 2–20% more supporting reads than the two FASTQ-based methods (RepeatHMM and Tandem-genotypes) on 10 samples and 23% more supporting reads than STRique. For normal STR alleles, DeepRepeat detects comparable number of supporting reads as RepeatHMM, and more supporting reads than Tandem-genotypes and STRique. The more supporting reads detected by DeepRepeat may explain why DeepRepeat works better in low coverage data (which is the case when performing whole-genome sequencing), as it is capable of using more information than competing approaches. In summary, unlike the other STR-detection methods, DeepRepeat identifies more repeat reads, allowing better quantification of repeat counts from raw electric signal intensity data, which is critical for whole genome data analysis with low coverage.

### Evaluation on synthetic sequences with GGGGCC repeats

DeepRepeat was next assessed on synthetic GGGGCC repeat sequences, where five synthetic molecules were sequenced [37] after polymerase chain reaction (PCR). The five sequences have 8, 32, 50, 56, and 76 GGGGCC repeat units, respectively. The nanopore data of the first three sequences were used for training DeepRepeat. Please note that only mapped nucleotides are used in the training process and the training data consists of 28,512 long reads from the 3 samples. That is, only 3 GGGGCC repeat units in the reference sequence and their flanking sequences are used in training process and the training data is comparatively small for DeepRepeat.

The nanopore data for the last two sequences were used to evaluate DeepRepeat. The estimation of repeat counts by RepeatHMM, DeepRepeat and Tandem-genotypes is shown in Additional file 1: Fig. S6 (STRique is not used in this comparison since it was trained and optimized on these synthetic sequences). In Additional file 1: Fig. S6a and S6b, all three methods have peaks closed to 56 and 76, demonstrating accurate estimation of repeat counts. Other repeat counts are also detected around 32 and 50 (Additional file 1: Fig. S6a), and 32, 50 and 56 (Additional file 1: Fig. S6b). This could reflect inaccurate demultiplexing of the original data, since all synthetic sequences were barcoded for mixed nanopore sequencing and then demultiplexed into different groups [37]. In summary, this analysis shows that DeepRepeat can be trained on a relatively modest training data (small number of repeat units) to achieve reasonable performance on alleles with much larger repeat counts.

### Evaluation of DeepRepeat on 57 manually curated STRs in the human genome

To obtain a well-trained model of DeepRepeat on a genome-wide scale, we trained it on all STR regions in GRCh38 and tested on 57 manually curated STRs. To generate the whole-genome training data we first downloaded whole-genome annotations on STRs from the UCSC genome browser [43], and then refined the whole genome repeat regions to obtain non-overlapping and perfect STR loci for GRCh38. Subsequently, we grouped whole-genome STRs according to their repeat units, and trained each model as a group. Taking trinucleotide repeats for example, there are 10 groups for 60 trinucleotide repeat motifs (excluding mononucleotide repeats). The grouping is necessary because an STR region may contain multiple motifs at the same time. For instance, in a STR region of **CAG**C**AGC**A**GCA**G and its complementary **CTG**C**TGC**T**GCT**G, the three motifs CAG, AGC, and GCA all occur, while CTG, TGC, and GCT all occur in the complementary strand. Thus, the six motifs are assigned as a group within a single DeepRepeat model. The example of group information used is provided in Additional file 1: Table S3.

**Fig. 5:**
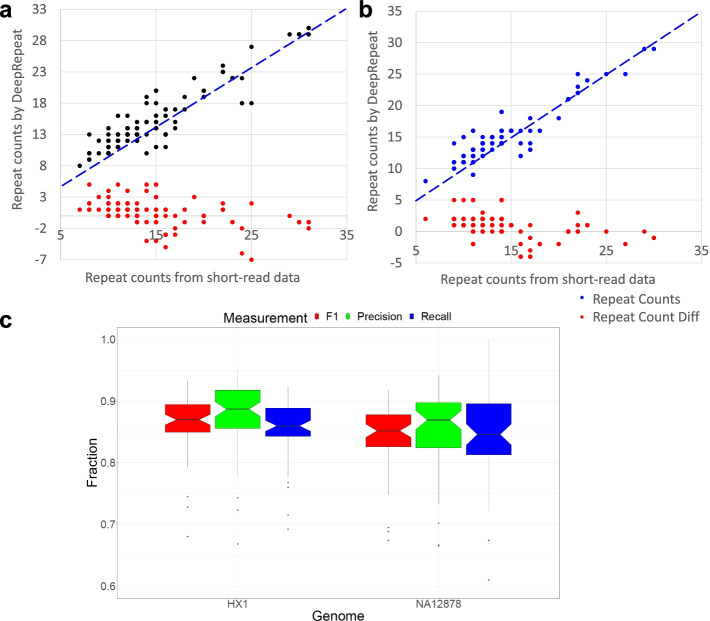
The performance of DeepRepeat with cross-genome testing strategy. **a** Repeat counts inferred by DeepRepeat on nanopore data against by HipSTR on high-coverage short-read data for NA12878 while DeepRepeat is trained on HX1. **b** Repeat counts inferred by DeepRepeat on nanopore data against by HipSTR on high-coverage short-read data for HX1 while DeepRepeat is trained on NA12878. **c** The whole genome performance for STR-nucleotide prediction by DeepRepeat. “Repeat Count Diff”: counts predicted by DeepRepeat minus the corresponding counts predicted by HipSTR

We next used 2 whole genome nanopore sequencing data for human individuals, NA12878 [44] and HX1 [45], to assess the accuracy of DeepRepeat. We trained DeepRepeat on NA12878 and then tested the trained model on HX1 with 57 manually curated STRs, or vice versa. The 57 manually curated STRs represent well-known disease-associated repeats or STRs widely used in forensic analysis. Since both NA12878 and HX1 are free from repeat expansion disorders, the repeat counts inferred from high-coverage short reads data of NA12878 and HX1 would be a reliable benchmark. Furthermore, both genomes have very high coverage short-read data (~300X for NA12878 and ~142X for HX1) generated by Illumina sequencing, and thus, the repeat counts detected on short-reads provide an independent evaluation of the DeepRepeat output. As shown in Fig. 5a, b, repeat counts for the 57 manually curated STRs estimated by DeepRepeat were compared against the repeat counts on NA12878 and HX1 short-read data inferred by HipSTR [26]. On the NA12878 genome, the repeat counts inferred by nanopore data and short-read data have a correlation coefficient 0.92 with an average absolute difference of 1.64, while on the HX1 genome, the correlation coefficient is 0.91 with an average absolute difference of 1.62. This indicates that the majority of repeat counts inferred by DeepRepeat on nanopore data are similar to the repeat counts estimated from high-coverage short-read data. In summary, we demonstrated the accuracy of DeepRepeat, even for relatively short STRs.

### Evaluation of DeepRepeat on nine STR loci in NA12878 with Sanger sequencing

To further evaluate DeepRepeat, we selected nine STR loci (five trinucleotide STRs and four tetranucleotide STRs) and conducted Sanger sequencing on NA12878. As shown in Table 1, the length of nine STR regions ranges from 63 to 113bp in the reference genome of GRCh38/hg38, and the repeat counts detected from Sanger sequencing range from 14 to 27. We then ran HipSTR on ~300X short-read data, and DeepRepeat on 38X long-read data of NA12878 to determine the repeat counts of the nine STR loci (we manually checked the repeat count distribution for each locus to infer repeat counts, because some loci have limited supporting long reads).

**Table 1:** Nine repeat loci with the repeat count detection by Sanger sequencing, HipSTR on 300X short-read data and DeepRepeat/Tandem-genotypes/RepeatHMM on 38X long read data for NA12878

Repeat loci	Motif	GRCh38 length	Sanger		HipSTR		DeepRepeat		Tandem-genotypes		RepeatHMM	
chr12:4702128-4702202	CCA	74	23	24	25	25	27	27	26	27	24	24
chr17:10995712-10995775	CTG	63	19	20	20	20	20	20	20	22	19	20
chr16:73546662-73546741	TGC	79	25	26	24	24	27	27	24	24	21	23
chr7:152384548-152384614	CAC	66	19	22	18	20	20	24	22	25	20	23
chr1:161051967-161052060	CAC	93	21	26	N.A.	N.A.	19	24	21	26	21	26
chrX:55366379-55366473	TATC	94	22	23	23	23	24	24	23	23	23	24
chr10:107609688-107609784	ATCT	96	18	21	19	22	19	22	19	23	19	22
chr6:145272942-145273029	AGAT	87	14	18	14	18	14	18	14	17	14	19
chr4:42555085-42555198	TATC	113	26	27	26	27	28	28	27	28	27	27

**Fig. 6:**
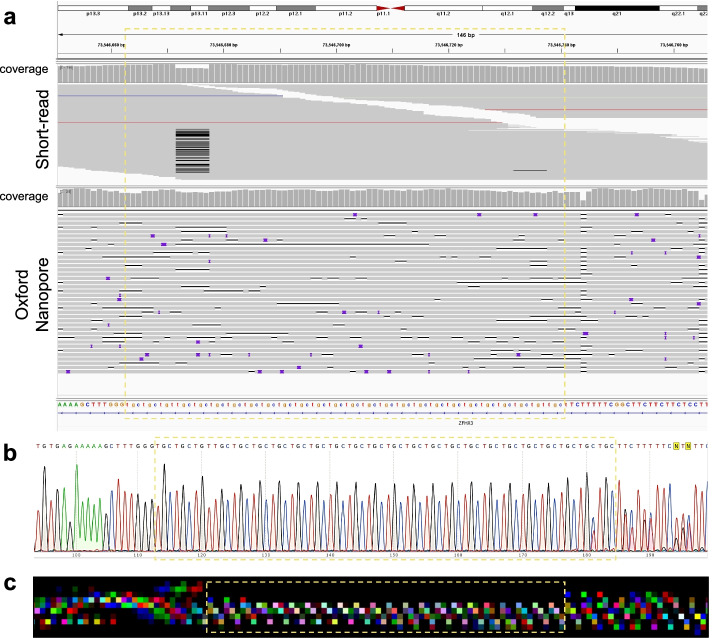
The TGC repeats at chr16:73,546,662–73,546,736 and the upstream/downstream regions. **a** In IGV plots with both short-read (upper) and long-read (lower) sequencings. **b** Sanger sequencing. **c** An example of RGB images generated for this region. The boxes in yellow indicate the repeat region

We also run Tandem-genotypes and RepeatHMM and summarized all results in Table 1. Since the nine STR loci do not have longer than 150 bp repeat regions, RepeatHMM and Tandem-genotypes also achieved good performance on several loci, and both DeepRepeat using long reads and HipSTR using short reads detect similar repeat counts as Sanger sequencing (Table 1). For example, the TGC repeat at chr16:73,546,662–73,546,741 has two alleles with repeat counts of 22 and 24 (as shown in Fig. 6 and confirmed by Sanger sequencing), while HipSTR with 300X short reads detects 23 repeats for both alleles. DeepRepeat detects the two alleles with 22 and 25 repeat counts. We also found that short-read sequencing data contain many reads that cannot completely span the repeat, and the short reads that do span the repeat have a 6 bp deletion, which may explain why HipSTR, even with 300X short reads, failed to detect the larger repeat allele (24 repeats). In contrast, DeepRepeat detected the two alleles and was consistent with the Sanger sequencing results in Fig. 6b. An example image in Fig. 6c shows a clear TGC repeat pattern.

**Fig. 7:**
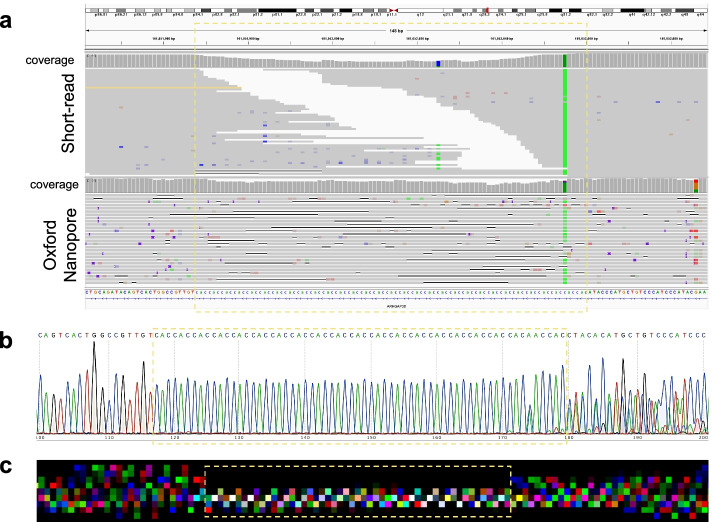
The CAC repeats at chr1:161,051,967–161,052,060 and the upstream/downstream regions. **a** In IGV plots with both short-read (upper) and long-read (lower) sequencings. **b** Sanger sequencing. **c** An example of RGB images generated for this region. The boxes in yellow indicate the repeat region

HipSTR also failed to detect the CAC repeats at chr1:161,051,967–161,052,060 (Table 1). We thus investigated the CAC repeat further: Fig. 7a clearly shows that there are no short reads spanning the repeat region despite that the overall coverage of short reads for NA12878 is 300X. In contrast, DeepRepeat is able to generate repeat counts for the two alleles which is consistent with the Sanger sequencing data (21 and 26 repeats for the two alleles). Figure 7b and an example image generated by DeepRepeat in Fig. 7c shows a clear CAC repeat pattern. Thus, DeepRepeat provides an accurate estimation of repeat counts. In Fig. 7a, there are notable indels in the repeat region, and these are expected: the reference genome for this region has 31 repeats while the Sanger sequencing data shows 21 and 26 repeats for the two alleles in this sample (Fig. 7b).

Please also note that in these relatively short STR regions, HipSTR, RepeatHMM, and Tandem-genotypes can achieve better estimation on some loci, such as chr12:4,702,128–4,702,202 and chr4:42,555,085-42,555,198 (Table 1). However, DeepRepeat achieves better performance on long STR regions as demonstrated before.

### Cross-genome evaluation of DeepRepeat for base-level repeat prediction

We next evaluated DeepRepeat for base-level repeat prediction on two whole-genome datasets. This evaluation is not to quantify repeat counts, but to assess if we can predict whether a given base is within a repeat or not. We cannot compare to other methods since they do not generate this type of prediction. For this analysis, we assumed that the labeled training data of STR loci and non-repeats in the reference genome are correct. Then, for each motif group, we used the DeepRepeat model trained on NA12878 to predict STR bases in HX1, or vice versa. Subsequently, we calculated precision, recall and F1 for each group where training data for a training group has more than 100 k training nucleotides. Groups with less training data are not considered since the training might not be enough to build a useful model. The results of repeat-nucleotide prediction are shown in Fig. 5c. The results show that DeepRepeat achieves a median recall of 0.878, a median precision of 0.856, and a median F1 of 0.868 on HX1. Also, we found a median recall of 0.861, median precision of 0.841 and median F1 of 0.842 on NA12878. For those genome-scale repeat regions and their flanking regions, ~ 89% nucleotides are correctly aligned for both HX1 and NA12878 with ~ 11% errors (including mismatches and indels with size less than 4 bp or the length of a repeat motif). Long indels may be repeat expansion and thus are not considered as errors. Since many sequence-based methods heavily relied on alignment for repeat size estimation, 89% would be their capped performance. The good performance of DeepRepeat demonstrates that DeepRepeat can accurately identify STR regions at a genome-wide scale.

### Comparison of running time

Here, we investigated the running time of DeepRepeat and STRique on 33 downsampled *HTT* data as previously described, and found that DeepRepeat is tens of times faster than STRique: on a computing node with 24 CPU cores (Intel(R) Xeon(R) CPU E5-2680 v3 @ 2.50GHz) and 128 GB memory, DeepRepeat used 120.6 seconds, while STRique used 3,624.8 seconds. Please note that both STRique and DeepRepeat process nanopore signals so that they are directly comparable.

In addition, we compared the running time of DeepRepeat against a sequence-based method RepeatHMM. RepeatHMM is chosen because it achieves the best performance in an independent critical assessment [46] and it is among a few methods with genome-scale estimation function, yet many other computational tools can only handle candidate regions with known patterns. In our experiment, RepeatHMM uses an Univa Grid Engine computing cluster to process multiple jobs at the same time, while DeepRepeat is run on the same cluster system. The cluster system has tens of shared computing nodes, each of which has 24 CPU cores (Intel(R) Xeon(R) CPU E5-2680 v3 @ 2.50GHz) and 128 GB memory. After that, the CPU time is summed for comparing RepeatHMM and DeepRepeat. On NA12878, DeepRepeat uses ~ 3.5 folds (~ 300 hours) of CPU running time more than RepeatHMM (~ 84 h), while the running time is ~ 5 fold higher on HX1 (~ 418 h). This is to be expected, because DeepRepeat use nanopore signals as input while RepeatHMM takes base-called long reads as input. Nanopore signal data is tens of times larger than long reads: long reads on HX1 and NA12878 are about 287Gb and 215Gb in uncompressed FASTQ format, while the nanopore signals is ~ 7 Tb in fast5 format.

In summary, although this is not a direct comparison of running time due to various programming languages for implementation and two different data types, DeepRepeat is efficient to process signal data for a genome-scale scanning.

## Discussion

In this study, we used a deep convolutional neural network to detect STRs from ionic signals in nanopore sequencing data by converting the STR detection problem into an image recognition problem, where the self-similarity of directly adjacent repeat units results in characteristic patterns in reconstructed images. We converted the signal distribution of repeat units into color images and then used the DeepRepeat framework to learn patterns of repeat images from non-repeat images. Through extensive testing on real data sets, DeepRepeat demonstrates excellent performance to infer STRs, especially very long STRs. DeepRepeat together with nanopore signal data thus provides a novel method to detect STRs, addressing the challenge of high basecalling error rates on STRs inherent in nanopore data and the poor alignment in low-complexity regions of the human genome. Depending on the specific application scenarios, we believe that DeepRepeat can complement other repeat detection approaches that rely solely on sequence data and together characterize both novel and known repeats in different regions of the human genome from long-read whole-genome sequencing data.

In particular, we used DeepRepeat to detect long STRs for CHM13 and for 11 samples with Huntington’s disease with repeat counts ranging from 39 to 72 and found that DeepRepeat is powerful to detect larger repeat counts. Huntington’s disease is a well-known disorder caused by trinucleotide (CAG) repeat expansion, and its severity and earlier age onset exhibit a clear association with larger repeat counts [2] (usually exceeding 39 repeat counts). As nanopore technique advances, sequencing cost has been substantially decreased, and nanopore sequencing is becoming a feasible way to sequence a large set of patients targeting a specific region of interest for disorders such as Huntington’s disease. In our testing, DeepRepeat achieves excellent performance with more supporting reads for larger repeat counts: this is particularly critical because in sequencing or PCR, reads with larger repeat counts are usually sequenced with less supporting reads than those with smaller repeat counts; more supporting reads detected by DeepRepeat for larger repeat counts means that DeepRepeat can use more information that are missed by other methods. DeepRepeat thus provides an efficient and effective way to analyze a large set of nanopore sequencing data for disease identification and risk prediction. Moreover, users can extend well-trained DeepRepeat model with more training on their own data set for a specific repeat locus to achieve improved detection rate. This is critically useful for a large-scale study of certain expansion disorders such as Huntington’s disease.

We also wish to discuss the differences of nanopore sequencing with competing approaches for repeat quantification. For telomeric repeats, qPCR-based methods are among the most widely used approaches; however, they cannot distinguish telomeric repeats from different chromosomes and thus the quantification is an average repeat count of all telomeres. DeepRepeat can detect the repeat count of each telomere, based on the mapping of nanopore reads. The cons are that nanopore sequencing has much higher cost than traditional qPCR methods, and it is difficult to perform amplicon-based or capture-based nanopore sequencing for telomeric regions, and that it requires more computational resources for data analysis on whole-genome data. The traditional method for Huntington’s disease repeat quantification is PCR-based fragment analysis. Long-read sequencing has the advantage of high throughput. In our internal experiments, we can multiplex and sequence 500 HD samples per MinION flow cell, by using barcoded primers and high-fidelity long-range PCR. The PCR product can be several kb. In addition, we can call haplotyped genetic variants from long reads, including repeat expansions and SNPs. This has strong clinical implications since understanding the haplotype structure of a repeat region allows design of allele-specific therapeutic approaches. Traditional methods such as Sanger sequencing cannot provide haplotype information because they can only assay a 500–1000 bp region, despite higher accuracy in SNP and indel detection.

In this study, we provide different well-trained repeat models according to the length of the repeat motifs and the motif groups. DeepRepeat will automatically chooses the model according to the input repeat motif. In the future, a generic DeepRepeat model may be generated for all possible STR motifs, since DeepRepeat is based on self-similarity of neighborhood signals. However, suitable training datasets are needed. Right now, we generated training data from NA12878 and HX1 according to GRCh38, but the occurrence instances of different motifs are highly unbalanced, and longer STR motifs have much less occurrences. Thus, it is not straightforward to have a balanced dataset to train a generic model, yet motif-specific trained models are used in the current study.

We also recognize that DeepRepeat has several limitations. DeepRepeat is a supervised deep learning method and requires a large amount of labeled training data. Here, we generated training data according to perfect STR regions in the whole genome of GRCh38. However, with larger STR motifs, there are fewer STR regions in whole-genome data for training. As such, we have provided well-trained DeepRepeat models for STR detection with repeat unit size up to 6 bp. According to the input motif, a proper well-trained DeepRepeat model is automatically selected; but users might need to train a DeepRepeat model if the repeat motif of interest (for example, > 6 bp repeat motifs) is not provided here. A second limitation is that DeepRepeat is based on nanopore data, and it may not accurately identify mono-nucleotide repeats (homopolymers) such as mono-A/C/T/G repeats. Furthermore, DeepRepeat is trained to identify STR regions based on self-similarity in a local window. If two STR regions are separated by a few bp, DeepRepeat may call them as a single rather than two separate STR regions. Similarly, DeepRepeat is not designed to detect mutations or extensive polymorphisms in STR regions. Additionally, DNA modifications such as base methylations may alter nanopore signals. Nonetheless, the limitations of DeepRepeat noted here may be overcome as more and more nanopore data become available.

## Conclusions

DeepRepeat is a novel computational method to detect STRs from nanopore sequencing data through direct analysis of electric signals rather than basecalled reads. In particular, DeepRepeat allows the analysis of STRs within or close to very low-complexity genomic regions, such as telomeric regions, where other methods fail or have poor performance. Additionally, DeepRepeat uses more long reads on STRs that are unusable by other algorithms, partially due to high basecalling errors in STR regions. This is important for pathogenic or likely pathogenic STR alleles because these alleles are usually longer and have fewer supporting long reads in sequencing data. Finally, the idea of converting signals to images in DeepRepeat provides an intriguing solution for many signal processing problems encountered in genomics settings with a deep learning framework.

## Methods

In this section, we discuss several types of data used in the study (STR regions, nanopore long-read data and short-read data), the framework of DeepRepeat, the methods to train and test DeepRepeat, the process of obtaining STR identification in CHM13, and wet-lab validation of several STRs.

### STR regions used in DeepRepeat

#### Whole genome STR regions for genome-scale DeepRepeat models

To obtain enough STR regions for training DeepRepeat models, whole genome STR regions defined by TRF (tandem repeat finder [47]) were downloaded from UCSC genome browser [43]. In the original file, there were 432,604 repeat records in GRCh38 with various repeat motifs. However, these repeat records cannot directly be used for training or testing, because many of these repeat records substantially overlapped with different repeat units or were not exact repeat regions. Thus, the repeat records were refined by (i) using semi-global alignment to align repeat records in the original file against a simple repeat sequence of the similar length and (ii) obtaining matched STR regions which were 3+ times longer than repeat units (or 12+ bases long for dinucleotides). Matched STR regions with small unmatched gaps were merged for a single STR region. As a result, 280,343 STR regions were obtained for training or testing DeepRepeat.

#### Manually curated STR loci in GRCh38 to test DeepRepeat for estimating repeat counts

To evaluate DeepRepeat, 57 STR loci were manually curated, including 2 di-nucleotide repeat, 20 tri-nucleotide repeats, 33 tetra-nucleotide repeats, and 2 penta-nucleotide repeats. The detail is given in Additional file 1: Table S4. Those STRs are associated with many neurological disorders or used for forensic analysis. The repeat start position and end positions of repeats in GRCh38 were manually checked for further analysis.

### Sequencing data for training and testing DeepRepeat

#### Nanopore data of the *HTT* gene from Huntington samples

To test DeepRepeat on pathogenic STR alleles, the *HTT* region on 11 samples with Huntington diseases and NA12878 (control) were sequenced on a nanopore GridION sequencer using the following process: We used a high-fidelity PCR enzyme (PrimeSTAR GXL DNA Polymerase, TaKaRa) to amplify the region flanking the CAG repeat region in the *HTT* gene. The amplicon region was chr4:3069608-3075517 (GRCh38 coordinates). The forward primer was AAAACGAGGGTTGTCAAAGACCCCA, and the reverse primer was GAGGGAAGTGGCACTGAGCAAATCT. For each sample, a unique 16 bp barcode was added to the 5′-end of each forward and reverse primer. The PCR condition was 98 °C for 10 s, 68 °C for 10 min, 30 cycles (2-step PCR). The PCR products were pooled and purified using AMPure XP beads. The library was prepared using the ligation sequencing kit (SQK-LSK108, Oxford Nanopore sequencing) according to the manufacturer’s instructions. The library was sequenced in the FLO-MIN106 (R9.4) flow cell in the GridION Nanopore sequencer. Briefly, 2.0 μg pooled and purified PCR products were used as the input DNA of each library. End repair and dA-tailing was performed using NEBNext Ultra II End Repair/dA-tailing Module (catalog No. E7546). In all, 7 μl Ultra II End-Prep buffer, 3 μl Ultra II End-Prep enzyme mix were added to the input DNA. The total volume was adjusted to 60 μl by adding nuclease-free water (NFW). The mixture was incubated at 20 °C for 5 min and 65 °C for 5 min. A 1 × volume (60 μl) AMPure XP clean-up was performed and the DNA was eluted in 31 μl NFW. One microliter of the eluted dA-tailed DNA was quantified using the Qubit fluorometer. A total of ≥ 1.0 μg DNA should be retained if the process was successful. Adaptor ligation was performed using the following steps. Twenty microliter Adaptor Mix (ONT, SQK-LSK108 Ligation Sequencing Kit) and 50 μl NEB Blunt/TA Master Mix (NEB, catalog No. M0367) were added to the 30 μl dA-tailed DNA. The mixture was incubated at room temperature for 10 min. The adaptor-ligated DNA was cleaned up using 40 μl of AMPure XP beads. The mixture of DNA and AMPure XP beads was incubated for 5 min at room temperature and the pellet was washed twice by 140 μl ABB (SQK-LSK108). The purified-ligated DNA was resuspended in 15.5 μl ELB (SQK-LSK108). A 1-μl aliquot was quantified by fluorometry (Qubit) to ensure ≥ 500 ng DNA was retained. The final library was prepared by mixing 35.0 μl RBF (SQK-LSK108), 25.5 μl LBB (SQK-LSK108), and 14.5 μl purified-ligated DNA. The library was loaded to R9.4 flow cells (FLO-MIN106, ONT) according to the manufacturer’s guidelines. GridION sequencing was performed using default settings for the R9.4 flow cell and SQK-LSK108 library preparation kit. The sequencing was controlled and monitored using the MinKNOW software developed by the manufacturer. In total, 5.24 Gb data was generated.

Then, Albacore v2.3.1 was used to conduct basecalling with events, which was required by DeepRepeat (note that while the Albacore software is obsolete for basecalling, we only used it to infer an event table, and we did not use basecalled sequences). After demultiplexing, the long reads were downsampled for each sample. As a result, there were ~4000 long reads for eight samples, ~ 2800 long reads for one sample, ~ 2400 long reads for two samples, and ~2000 long reads for one sample. The majority of long reads had ~ 6000 bp as expected. The CAG repeat counts for each sample were provided in [48] where the repeat counts for 12 samples ranged from 17 to 72.

#### Nanopore data for GGGGCC repeats

Giesselmann et al. recently published a nanopore data set on synthetic DNA molecules with GGGGCC repeats [37] after polymerase chain reaction (PCR). Among them, 5 synthetic DNA had enough reliable data with 8, 32, 50, 56, and 76 GGGGCC repeats, respectively. The nanopore data for these synthetic DNA was basecalled with event information using Albacore version 2.3.1. DeepRepeat used the event information, but not basecalled sequences for repeat detection.

#### Whole genome nanopore data

Two published nanopore data of human DNA genome, NA12878 and HX1, were used in training and testing DeepRepeat. The fast5 files in the two genomes were downloaded from https://github.com/nanopore-wgs-consortium/NA12878/blob/master/Genome.md and PRJNA533926 at NCBI and basecalled with Albacore version 2.3.1 with event information. Then, there were 12,743,232 passed long reads for NA12878 with ~38X coverage and 11,210,579 passed long reads for HX1 with 51X coverage.

#### Short reads data to infer benchmark repeat counts for evaluating DeepRepeat

High-coverage short-read data of NA12878 and HX1 were used to infer repeat counts of manually curated STR loci for evaluating DeepRepeat. For NA12878, a BAM file of ~300X coverage of 150 bp HiSeq2500 short reads data was downloaded from NIST, and for HX1, the short reads data published in [49] were used with ~ 1.42 million paired reads and ~142X coverage of ~ 429 Gigabases. The short reads of HX1 were aligned with GRCh38 using minimap2 [41] with the parameters for paired short reads. HipSTR [26] was used to detect repeat counts of manually curated STRs in both NA12878 and HX1 with the minimum support reads of 10. Since short-read sequencing has less than 0.5% error rates, it is expected that the repeat counts of STR regions are accurate for testing DeepRepeat on long reads data.

### DeepRepeat framework

DeepRepeat is a STR detection tool using a deep convolutional neutral network on nanopore signals. The input is a reference genome, an aligned bam file and fast5 files with basecalled events; the output is a repeat count distribution for an individual. In DeepRepeat, there are several steps: converting nanopore signals to image representation, classifying repeats and non-repeats with a deep learning framework, and quantifying repeat counts for long reads with peak calling for an individual.

#### Image conversion

In DeepRepeat, events together with signals in fast5 files for each long read were used to generate image representation. Usually, each event, anchored to a nucleotide, was associated with a series of signals. Before the conversion, raw signals were first normalized for each long read using the method in reference [50]. The range of normalized signals is from − 5 to 5. After that, the normalized signals were discretized into 50 non-overlapped bins with bin step equal to 0.2, and the 50-bin vector of signals was used to represent each event: the value in a bin was the fraction of the number of signals falling into that bin over the number of signals of the event, and the fraction was normalized with the range from 0 to 255, similar to value range in color images. Then, given a repeat unit of the length equal to *R*, each nucleotide in the repeat unit can be represented by a 50-bin vector, and *R* vectors were consecutively stacked to generate a black-white image with 50 as height and *R* as width.

In a *L*-base long read of *n*_0_, *n*_1_, ⋯, *n*_*j* − *R*_, *n*_*j* − *R* + 1_, ⋯, *n*_*j*_, *n*_*j* + 1_, ⋯, *n*_*j* + *R*_, *n*_*j* + *R* + 1,_⋯*n*_*j* + 2*R*_, ⋯, *n*_*L*_, any black-white image of a *R*-mer at *n*_*j*_ and its upstream/downstream of images at *n*_*j* − *R*_ and *n*_*j* + *R*_ were considered as three channel of a color image. With this representation, if the three directly adjacent *R*-mers are repeats, it is expected that the non-zero values of the three channels in an image are at the same position in the color image. In an ideal case, the color image of three consecutive repeat units would only have *R* white dots, where the three channels all have 255 at the *R* positions and 0 at all other positions in repeat images (similar to what is shown in the left of Fig. 1). Impurities in the repeat unit (imperfect tandem repeats) can still be represented as a white line with some noises, such as gray dots within white dots. In contrast, images which are generated from non-repeat regions would have much less non-overlapped non-zero values for any two channels, and thus different colors are randomly dispersed in images (as shown in the left of Fig. 1.).

#### Deep learning classification

After converting signals into color images with three channels, the next step was to design a deep convolutional neural network (CNN) to learn the patterns in images from repeat regions against images from non-repeat regions. CNN is regularized multilayer perception network where hierarchical local patterns in images are learned from original images with several convolutions/pooling layers. A neuron in a convolutional layer learns knowledge only from a restricted region in the previous layer rather than all regions in the previous layer to avoid significant overfitting. As multiple convolutional/pooling layers are used, more abstract local patterns with a larger region are learned, and are used as input of a fully connected network, possibly with another full connection hidden layer, for final prediction. CNN has been successfully applied in image recognition and classification [51], and natural language processing [52], and achieved great improvement to reduce the classification errors [51, 52].

In DeepRepeat, two hidden convolutional and pooling layers were used with 32 4X1 filters and 64 3X1, respectively, as shown in Fig. 1. The convolutional and pooling layer transformed the images’ size from 50X *R* with 3 channels, to 25X *R* with 32 depth, and then to 13x *R* with 64 depth. The 3-dimension data was flattened to form a vector with 832* *R* values for the input of a full connection network where the hidden layer had 512 neurons and the final output had 4 possibilities: “not in a repeat,” “in a repeat,” “deletion in a repeat,” or “insertion in a repeat.” For example, as shown in Fig. 1, for a repeat motif with 3 bp, the first convolutional and pooling layer led to 25X3X32 matrices, while the second layer led to 13X3X64; after flattening, the fully connection network had 2496 values for the first layer and 512 for the second layer and then generated 4 possibilities. In the training process of DeepRepeat, the Adam optimizer algorithm [53] was used based on the loss function of softmax cross entropy between the prediction and the benchmark labels of each base.

#### Repeat count quantification

With a well-trained DeepRepeat model, STR prediction was made on all nucleotides in each long read, or only on those bases in long reads which were aligned against reference regions of interest. For the latter case, all fastq sequences of a set of fast5 files were aligned against GRCh38 using minimap2 [41] with the parameters for nanopore long reads. Then, for each long read aligned against a region of interest, consecutive STR prediction was predicted, and any of the consecutive STR regions were merged if the length of the STR regions is 3 times longer than the length of repeat units and if the distance between two close STR regions was shorter than the length of repeat units. The length of merged STR regions was divided by the length of repeat units to get the repeat count in this long read. In a similar way, repeat counts were estimated for all long reads aligned with the region of interest, and a histogram of repeat counts was generated where the entry was the repeat count and the entry’s value was the times of this repeat count detected in all long reads aligned against the region of interest.

With the assumption that each repeat count of true alleles followed a Gaussian distribution in the histogram of repeat count, Gaussian mixture model implemented in scikit-learn was used to call the peak for the repeat region of interest. To do that, each of different Gaussian components (ranging from 3 to 7) was evaluated in Gaussian mixture model 20 times, and a model was selected with the best Akaike information criterion. After that, the peaks were called with more supporting reads (this process was similar to the peak calling process in our previous work of RepeatHMM [29]). The output would be one repeat count number for homogeneous alleles and two repeat count numbers for heterogeneous alleles. If the data for a sample had low coverage, users are advised to check the histogram for repeat count inference rather than using Gaussian mixture model, since Gaussian mixture model requires enough data for accurate inference.

### Training and testing

#### Labeling converted images

After converting signals to color images, a critical issue in training DeepRepeat was to obtain a larger set of labeled instances of images for repeat regions and for non-repeat regions. Given a set of nanopore data and a list of STR regions of interest, the general process below was used to assign label (“in repeat” or “not in repeat” or “a deletion in repeat” or “an insertion in repeat”) to images. After fastq sequences of long reads were aligned with a reference genome (GRCh38 in this study), (i) if a nucleotide was aligned with any base in any repeat region, “in repeat” was assigned to the image centered at the nucleotide in the long read; (ii) if a nucleotide was a 1-bp insertion (deletion) in repeat regions, “an insertion in repeat” (“a deletion in repeat”) was assigned to the image centered at the nucleotide in the long read; (iii) if a nucleotide was clipped or inserted (larger insertions) starting at STR regions or aligned with STR regions which were not of interest, images are constructed without no label (not used for training or testing); and (vi) for other nucleotide in the long reads which are not aligned with any STR regions, images are constructed with the labels “not in repeat.” By default, nucleotides were considered if they are 1.5* *M* bp away from all STR regions of interest where *M* is the length of a STR region.

#### Motif groups for training DeepRepeat models

In DeepRepeat, STR motifs of repeat units are usually considered as a group rather than individually. This is because a STR region has different repeat motifs. For example, in a repeat template region “CAGCAGCAGCAG,” all three STR motifs “CAG,” “AGC,” and “GCA” occur; at the same time, for the complementary repeat region “CTGCTGCTGCTG,” all three STR motifs “CTG,” “TGC,” and “GCT” occur. Thus, the 6 STR motifs can be considered together as a group to have a single DeepRepeat training model. The smaller motif in an alphabetic order (AGC) was used to represent this motif group. There were two more advantages for this grouping strategy: more instances were available for training a DeepRepeat mode, and less well-trained DeepRepeat models were generated.

Using the grouping strategy above, there are several different motif groups for repeat units with various lengths. For example, for trinucleotide repeats, there are 60 possibilities (“AAA,” “CCC,” “TTT,” and “GGG” are not considered since they are mononucleotide repeats). The 60 possibilities can be categorized into 10 groups, and each group has 6 repeat motifs. The similar group strategy was used for dinucleotide repeats, tetra-nucleotide repeats, and penta-nucleotide repeats.

#### Training and testing process

The process for training and independently testing DeepRepeat was described below. (1) To test DeepRepeat on the *HTT* alleles of 11 samples with Huntington’s diseases and of NA12878, DeepRepeat was trained with 200 epochs on our in-house larger datasets of Huntington’s diseases for CAG repeats in the *HTT* gene in GRCh38. Estimated repeat counts by DeepRepeat was compared to true repeat counts to see the repeat count estimation performance of different methods. The true repeat counts for the 12 samples were obtained from [48]. (2) On the published nanopore data of GGGGCC repeat, since the sequences were synthetically generated, the data of the 3 sequences with the shorter repeat regions (8, 32, and 50 GGGGCC repeats) were used to train DeepRepeat, and the 2 sequences with longer repeat regions were used to test DeepRepeat. Please note that there were only 3 GGGGCC repeats in the reference sequence, and many inserted repeats were not used in training process for the 3 sequences; thus, the training instances were not large enough. (3) To test DeepRepeat on 57 manually curated STR loci and whole genome STRs for repeat nucleotide prediction, DeepRepeat was evaluated on the whole genome STRs under a cross-genome independent testing strategy: DeepRepeat was trained on NA12878 and tested on HX1, or vice versa. Since the whole genome STRs had different lengths of repeat units, DeepRepeat was trained for each motif group. For each motif group on a training data, at most 100 randomly selected repeats were used for each chromosome, when the size of training instances was much larger. In the training process, training instances were split into different batches with batch size of 512. To consider unbalanced instances of repeat images and non-repeat images, non-repeat images were split into different groups and each group had the similar size of repeat images. The learning rate was set to 0.005 at the first *n* iterations, to 0.001 before n*10 iterations, to 0.0005 before *n* ∗ 100 iterations and to 0.0001 after n*100 iterations. *n* usually was set to 5, but to 1 for some motif groups. The training stopped after 500 epochs. In the cross-genome independent testing, a well-trained DeepRepeat model for a motif group on a genome was used to make repeat prediction on the other genome. In the evaluation of repeat-nucleotide performance for whole genome STRs, only labeled repeat and non-repeat were used, since the alignment of matched bases was believed to be accurate. For 57 manually curated STRs, the estimated repeat counts were compared against repeat counts calculated from high-coverage short-read data. Given 0.05% error rate of high-coverage short-read data and the shorter repeat regions of the 57 STRs, the repeat count inference from short-read data would be good enough as benchmark to evaluate repeat count estimation by DeepRepeat.

### Quantification of telomere repeats in CHM13

We downloaded 30X nanopore raw signal data (fast5 files) from the GitHub repository of the Telomere-to-telomere consortium (https://github.com/marbl/CHM13). The fast5 files were basecalled using Albacore v2.3.4, and the basecalled reads were aligned to the CHM13 v1.1 assembly using minimap2. After removing secondary and supplementary alignments, we used DeepRepeat to quantify telomere repeat length by supplying the fast5 files and the bam file. Of note, Albacore was only used to infer event tables (Guppy v5 cannot generate event tables), and we did not use basecalled sequence for repeat detection.

### Detection of long STRs in CHM13

To obtain long STR regions to evaluate DeepRepeat, we run TRF to find tandem repeats from CHM13 v1.1 assembly sequence and required that the length of repeat units<=6 bp, length of repeat regions > 200 bp, and similarity between repeat copies > 85%. After that, we further removed STR regions that were within a 500 of another STR because many adjacent STRs have similar repeat motifs and it is hard to tell if they should be merged or not. In the final list, we have 439 long STR regions (Additional file 2: Table S1). We used the CHM13 v1.1 assembly as the reference genome and performed repeat detection using DeepRepeat, RepeatHMM and STRique.

### Validation of the STR loci in the NA12878 sample

To further evaluate DeepRepeat, nine STR loci were selected to conduct Sanger sequencing on NA12878. We designed PCR primers within ~ 400 bp of a STR region of interest and then used a high-fidelity PCR enzyme (PrimeSTAR GXL DNA Polymerase, TaKaRa) to amplify each of the target selected STR regions. The PCR products were purified using AMPure XP beads and sequenced by Sanger sequencing. The STRs were validated by counting the numbers of repeat units in the Sanger sequencing results.

## Supplementary Information

**Additional file 1.** Figures S1-S7 and Tables S2-S4.

**Additional file 2: Table S1**. STR regions in CHM13 for evaluation.

**Additional file 3: Table S5**. Sequencing error statistics of the G4C2 repeat region.

**Additional file 4.** Review history.

## Data Availability

The sequencing data of the *HTT* region for 11 cell lines with Huntington diseases and NA12878 is available at the NCBI Sequence Read Archive (SRA) under the study PRJNA678742 [54]. DeepRepeat is under GPL v3.0 license and is publicly available on GitHub (https://github.com/WGLab/DeepRepeat) [55], and the version used in this study is available at Zenodo (10.5281/zenodo.5880245). A detailed description of installing/running DeepRepeat and reproducible pipelines/datasets has also been documented in the GitHub repository.
