# Supplementary material for: DeepRepeat: direct quantification of short tandem repeats on signal data from nanopore sequencing
Source: Genome Biol. 2022 Apr 28;23:108. doi: 10.1186/s13059-022-02670-6 (PMC9052667; doi:10.1186/s13059-022-02670-6)
Supplement: Supplementary file 1 — Additional file 1. Figures S1-S7 and Tables S2-S4. [file 13059_2022_2670_MOESM1_ESM.docx]

**Supplementary Data**

**DeepRepeat: direct quantification of short tandem repeats on signal data from nanopore sequencing**

Li Fang^1,#^, Qian Liu^1,2,3,#,*^, Alex Mas Monteys^1^, Pedro Gonzalez-Alegre^1^, Beverly L. Davidson^1,4^, Kai Wang^1,4*^

^1^ Raymond G. Perelman Center for Cellular and Molecular Therapeutics, Children's Hospital of Philadelphia, Philadelphia, PA 19104, USA

^2^ School of Life Sciences, College of Science, University of Nevada, Las Vegas, Las Vegas, NV, 89154, USA

^3^ Nevada Institute of Personalized Medicine, College of Science, University of Nevada, Las Vegas, Las Vegas, NV, 89154, USA

^4^ Department of Pathology and Laboratory Medicine, Perelman School of Medicine, University of Pennsylvania, Philadelphia, PA 19104, USA

^#^ The authors wish it to be known that, in their opinion, the first two authors are regarded as joint First Authors. ^*^ To whom correspondence should be addressed. Email: qian.liu@unlv.edu (Q.L.), wangk@chop.edu (K.W.)

# Supplementary Figures


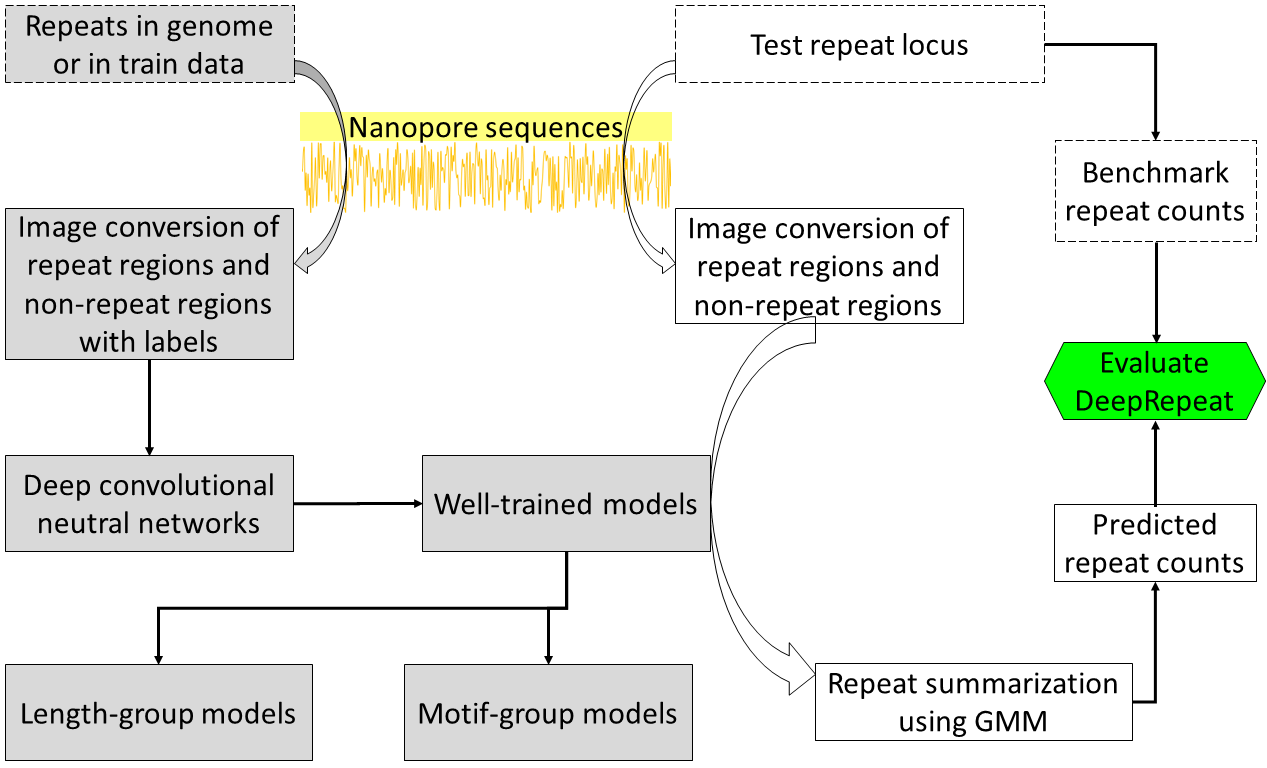


**Figure S1.** Overview of DeepRepeat and its testing. GMM: Gaussian mixture model. Shapes in grey is for training, while other for testing. Rectangles with dot borders is for input.


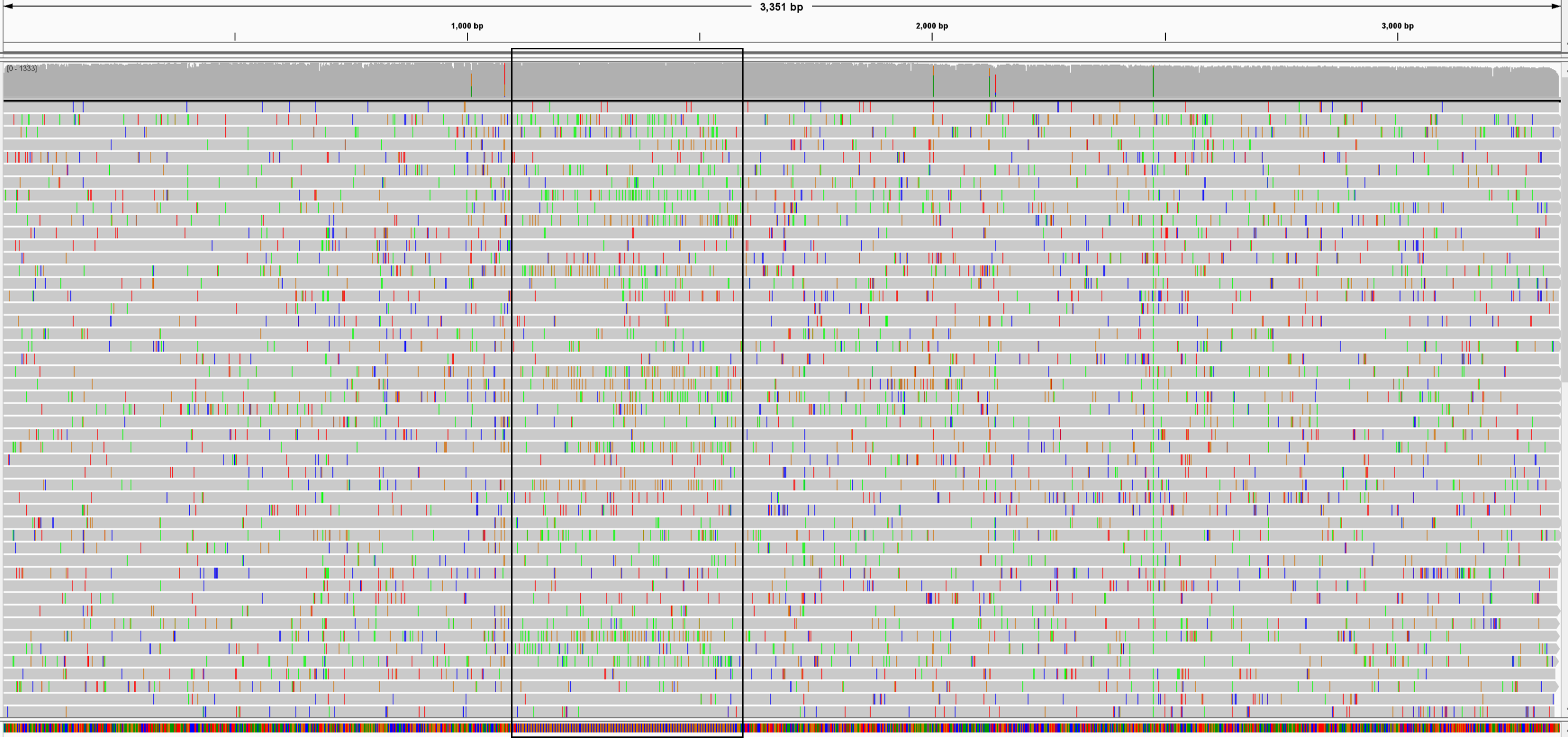


**Figure S2**. IGV screenshot showing sequencing errors of the 76-copy G4C2 repeat region (in the black rectangle). Secondary and supplementary alignments are filtered. Reads were basecalled by Guppy 5.


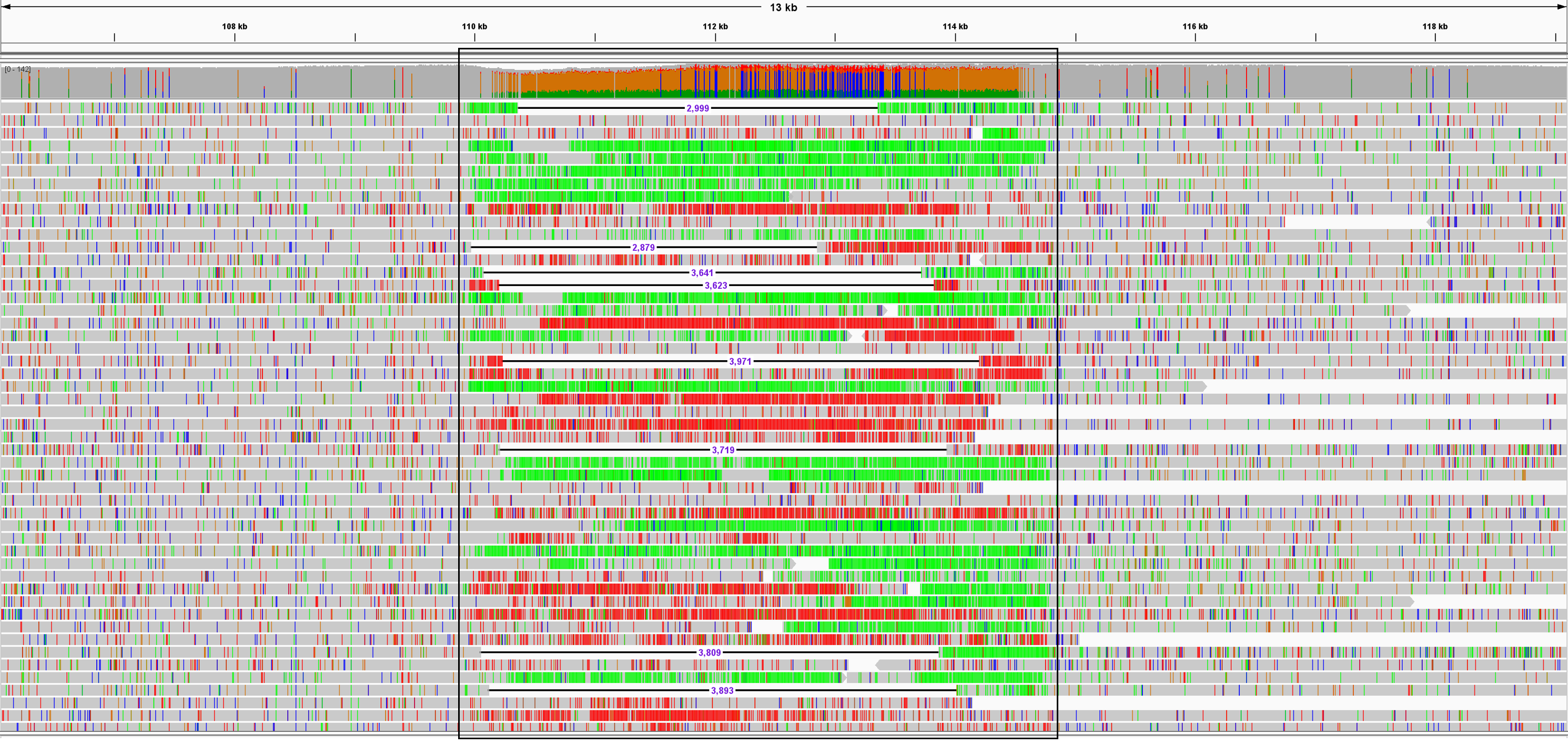


**Figure S3**. IGV screenshot showing sequencing errors of the 800-copy G4C2 repeat region (in the black rectangle). Secondary and supplementary alignments were filtered. Reads were basecalled by Guppy 5.

*
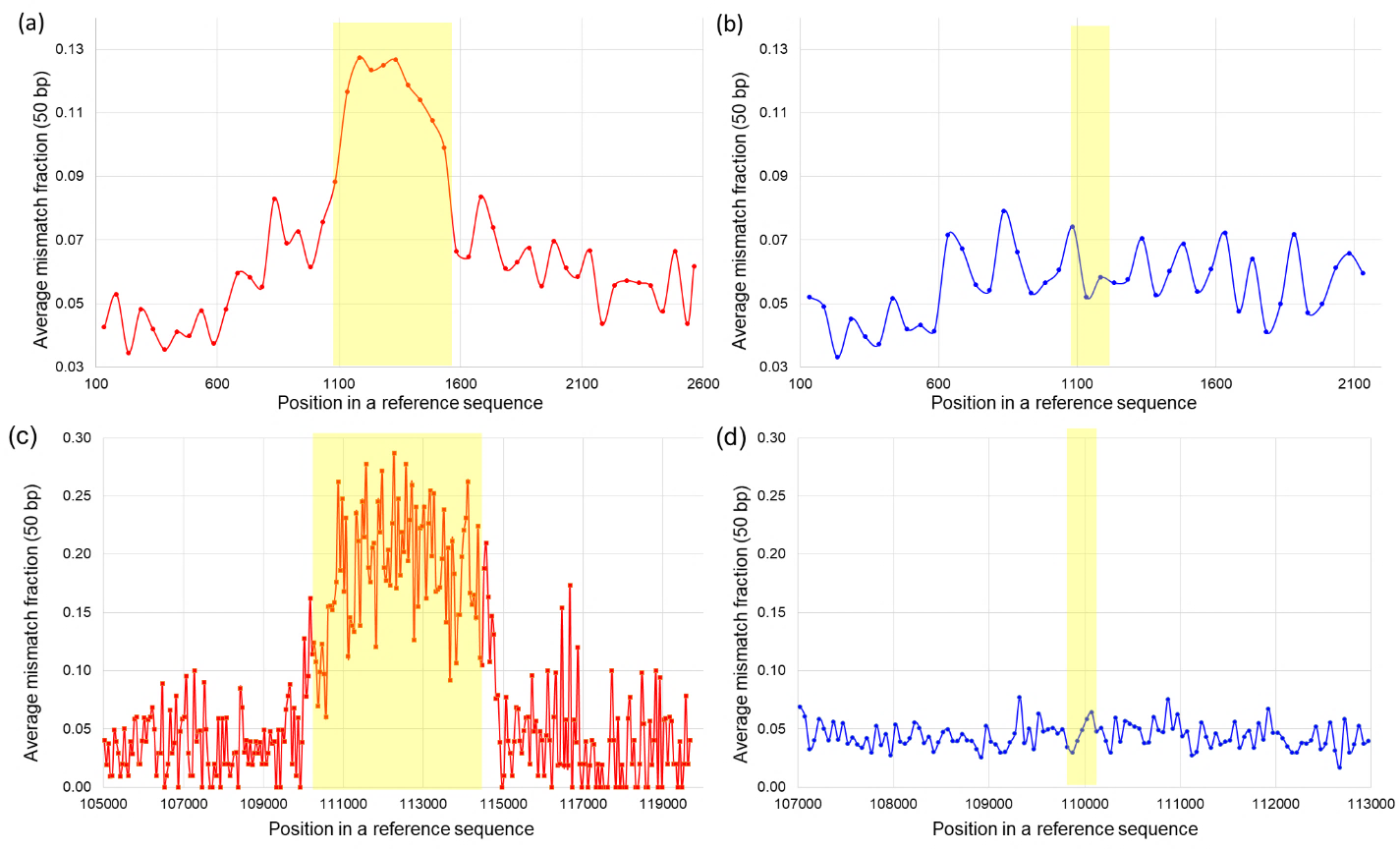
*

**Figure S4**. Mismatch error percentage for long reads with different repeat length (Repeat regions: shown in the rectangles filled in grey). G4C2 repeats: 76 repeat copies (a) and 8 repeat copies (b) for 2 synthetic sequences, and ~800 repeats (c) and 15 repeats (d) for a BAC clone. Each dot is for an averaged mismatch error of a 50bp region to reduce randomness. The long-read data were released by Giesselmann et.al. Nat. Biotech 2019 and basecalled with Albacore v2.3.


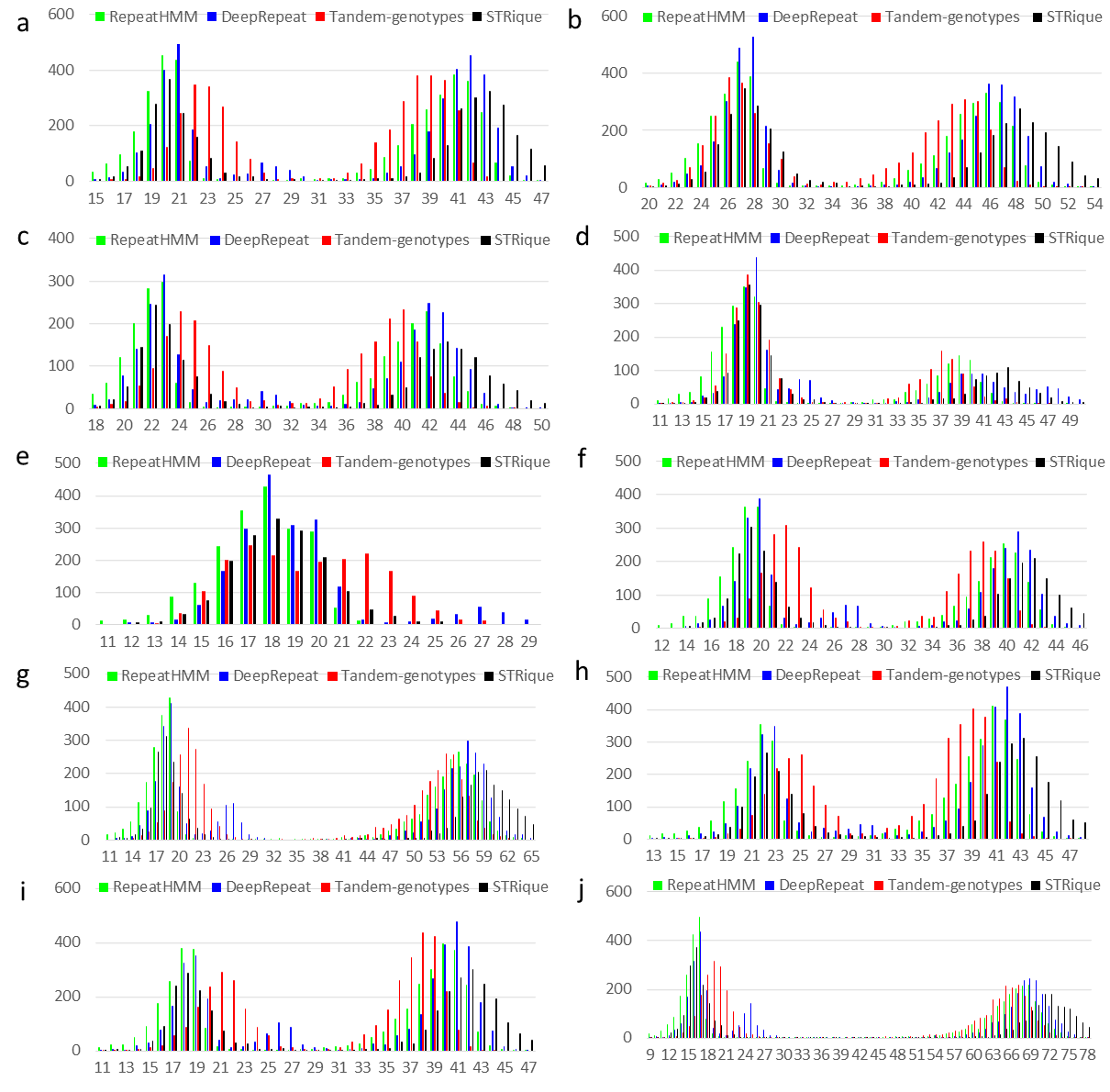


**Figure S5.** The distribution of estimated repeat counts on 11 Huntington samples with CAG repeats. (a): ND30047; (b): ND40534; (c): ND30015; (d): ND31551; (e): NA12878; (f): ND33947; (g): ND33392; (h): ND30016; (i): ND29970; (j): GM04723.


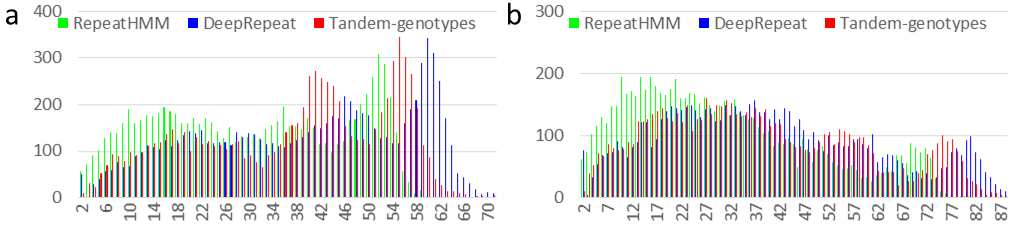


**Figure S6**. The distribution of estimated repeat counts on GGGGCC samples. (a) a sample which might contain 56 GGGGCC repeats, and (b) a sample which might contain 76 GGGGCC repeats. Please note that inaccurate demultiplexing leads to different peaks, since samples with repeat counts of 8, 32, 50, 56 and 76 are sequenced together.


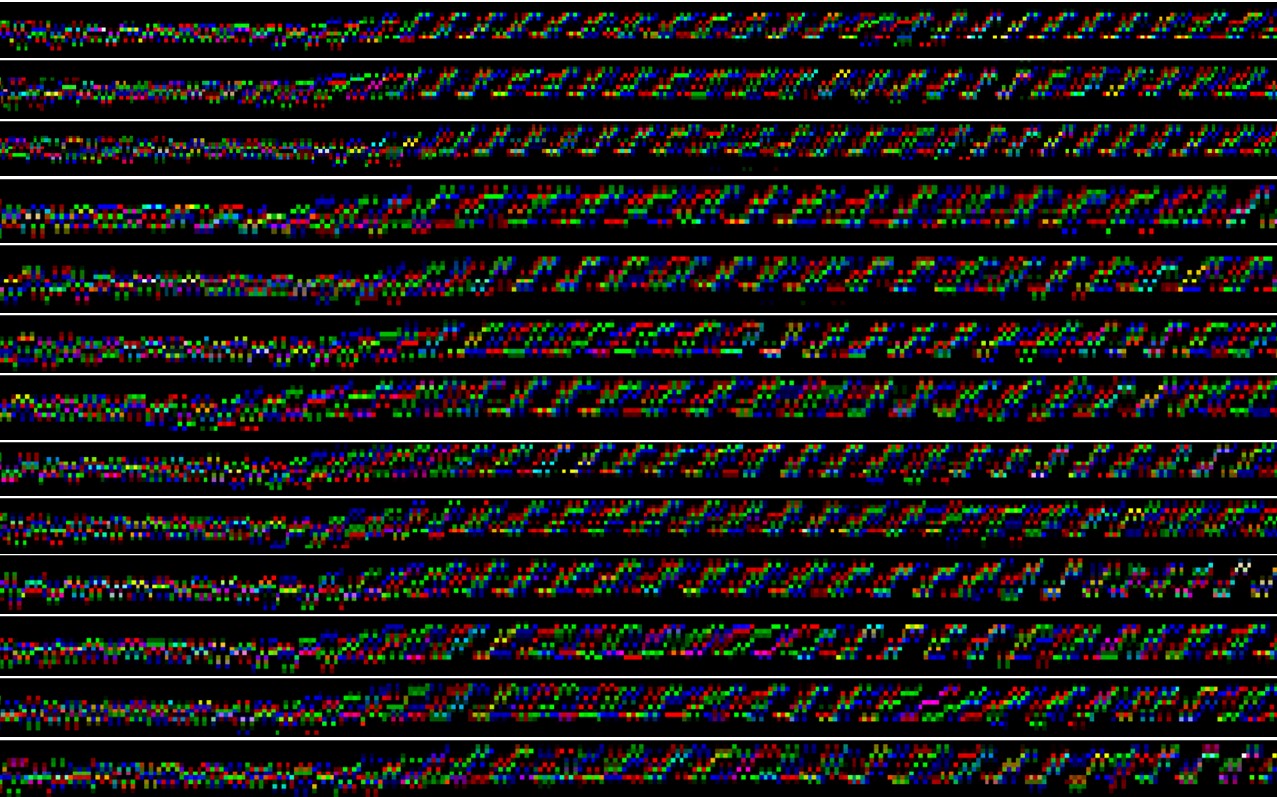


**Figure S7**. Image representation of DeepRepeat signals at the repeat regions (right) and the flanking regions (left) for the telomeric repeat (AACCCT, chr5:1-2297, CHM13 v1.1 assembly). Each row is for a long read, while each column is for a base. The repeats in all reads are visually represented as colored ribbon.

# Supplementary Tables

**Table S1**. STR regions in CHM13 for evaluation. The coordinates are based on the CHM13 assembly v1.1 (Table in a separate Excel file).

**Table S2**. The number of supporting reads for estimated repeat counts. Each row is for a HTT PCR data. The number of supporting reads for each method is calculated by the sum of the number of supporting reads for an estimated repeat count number ($ERC$) and its $k-1$ adjacent neighbor number of estimated repeat counts where $k=int(ERC)/20$. The last three columns are how much more percentage of the number of supporting reads DeepRepeat detects compared to other methods where R: RepeatHMM; T: Tandem-genotypes; S: STRique and D: DeepRepeat.

| **HTT data** |  | **Repeat**  **HMM** | **Tandem-**  **genotypes** | **STRique** | **DeepRepeat** | **(D-R)/D** | **(D-T)/D** | **(D-S)/D** |
| --- | --- | --- | --- | --- | --- | --- | --- | --- |
| ND30047 | Pathogenic  Repeat | 1,567 | 1,667 | 1,326 | 1,730 | 9.4% | 3.6% | 23.4% |
| ND40534 |  | 1,403 | 1,338 | 1,110 | 1,464 | 4.2% | 8.6% | 24.2% |
| ND30015 |  | 818 | 836 | 682 | 914 | 10.5% | 8.5% | 25.4% |
| ND31551 |  | 548 | 562 | 408 | 387 | -41.6% | -45.2% | -5.4% |
| ND33947 |  | 977 | 1,041 | 809 | 1,050 | 7.0% | 0.9% | 23.0% |
| ND33392 |  | 1,291 | 1,244 | 1,029 | 1,385 | 6.8% | 10.2% | 25.7% |
| ND30422 |  | 1,384 | 1,483 | 1,249 | 1,738 | 20.4% | 14.7% | 28.1% |
| ND30626 |  | 1,297 | 1,585 | 1,155 | 1,639 | 20.9% | 3.3% | 29.5% |
| ND30016 |  | 1,593 | 1,686 | 1,280 | 1,718 | 7.3% | 1.9% | 25.5% |
| ND29970 |  | 1,570 | 1,690 | 1,235 | 1,708 | 8.1% | 1.1% | 27.7% |
| GM04723 |  | 1,141 | 1,184 | 998 | 1,344 | 15.1% | 11.9% | 25.7% |
| ND30047 | Normal repeat | 1,214 | 934 | 890 | 1,078 | -12.6% | 13.4% | 17.4% |
| ND40534 |  | 1,157 | 1,000 | 891 | 1,231 | 6.0% | 18.8% | 27.6% |
| ND30015 |  | 642 | 607 | 587 | 689 | 6.8% | 11.9% | 14.8% |
| ND31551 |  | 965 | 978 | 904 | 947 | -1.9% | -3.3% | 4.5% |
| NA12878 |  | 1,082 | 666 | 898 | 1,074 | -0.7% | 38.0% | 16.4% |
| ND33947 |  | 794 | 835 | 762 | 884 | 10.2% | 5.5% | 13.8% |
| ND33392 |  | 891 | 870 | 814 | 915 | 2.6% | 4.9% | 11.0% |
| ND30422 |  | 772 | 755 | 671 | 830 | 7.0% | 9.0% | 19.2% |
| ND30626 |  | 836 | 892 | 820 | 1,078 | 22.4% | 17.3% | 23.9% |
| ND30016 |  | 902 | 679 | 674 | 802 | -12.5% | 15.3% | 16.0% |
| ND29970 |  | 1,014 | 794 | 753 | 872 | -16.3% | 8.9% | 13.6% |
| GM04723 |  | 1,000 | 873 | 889 | 947 | -5.6% | 7.8% | 6.1% |

**Table S3.** Example for the group of motifs for 2-mer, 3-mer and 4-mer. The group information of 5-mer and 6-mer can be similarly generated or using our script in DeepRepeat GitHub.

| Motif group | Motif list |
| --- | --- |
| AC | CA,AC,TG,GT |
| AG | GA,TC,AG,CT |
| AT | TA,AT |
| CG | GC,CG |
| AAC | CAA,ACA,AAC,TTG,TGT,GTT |
| AAG | GAA,AGA,TTC,AAG,TCT,CTT |
| AAT | TAA,ATA,TTA,AAT,TAT,ATT |
| ACC | CCA,CAC,ACC,TGG,GTG,GGT |
| ACG | CGA,GAC,GTC,ACG,TCG,CGT |
| ACT | CTA,GTA,TAC,TAG,ACT,AGT |
| AGC | GCA,AGC,TGC,CAG,CTG,GCT |
| AGG | GGA,TCC,CTC,GAG,AGG,CCT |
| ATC | TCA,TGA,ATC,ATG,CAT,GAT |
| CCG | GCC,CGC,GGC,CCG,GCG,CGG |
| AAAC | CAAA,ACAA,AACA,AAAC,TTTG,TTGT,TGTT,GTTT |
| AAAG | GAAA,AGAA,AAGA,TTTC,AAAG,TTCT,TCTT,CTTT |
| AAAT | TAAA,ATAA,AATA,TTTA,AAAT,TTAT,TATT,ATTT |
| AACC | CCAA,ACCA,CAAC,AACC,TTGG,GTTG,TGGT,GGTT |
| AACG | CGAA,ACGA,GAAC,GTTC,AACG,TTCG,TCGT,CGTT |
| AACT | CTAA,ACTA,GTTA,TAAC,TTAG,AACT,TAGT,AGTT |
| AAGC | GCAA,AGCA,AAGC,TTGC,CAAG,CTTG,TGCT,GCTT |
| AAGG | GGAA,AGGA,TTCC,CTTC,GAAG,AAGG,TCCT,CCTT |
| AAGT | GTAA,AGTA,CTTA,TTAC,TAAG,TACT,AAGT,ACTT |
| AATC | TCAA,ATCA,TTGA,AATC,ATTG,CAAT,TGAT,GATT |
| AATG | TGAA,TTCA,ATGA,ATTC,AATG,GAAT,TCAT,CATT |
| AATT | TTAA,ATTA,TAAT,AATT |
| ACAC | CACA,ACAC,TGTG,GTGT |
| ACAG | GACA,CAGA,AGAC,TGTC,ACAG,TCTG,GTCT,CTGT |
| ACAT | TACA,CATA,TGTA,ATAC,TATG,ACAT,GTAT,ATGT |
| ACCC | CCCA,CCAC,CACC,ACCC,TGGG,GTGG,GGTG,GGGT |
| ACCG | CCGA,CGAC,GACC,GGTC,ACCG,GTCG,TCGG,CGGT |
| ACCT | CCTA,GGTA,CTAC,TACC,GTAG,TAGG,ACCT,AGGT |
| ACGC | CGCA,GCAC,ACGC,GTGC,CACG,TGCG,CGTG,GCGT |
| ACGG | CGGA,GGAC,GTCC,CGTC,GACG,TCCG,ACGG,CCGT |
| ACGT | CGTA,GTAC,TACG,ACGT |
| ACTC | CTCA,GTGA,TCAC,ACTC,TGAG,AGTG,CACT,GAGT |
| ACTG | GTCA,CTGA,TGAC,AGTC,TCAG,ACTG,GACT,CAGT |
| AGAG | GAGA,TCTC,AGAG,CTCT |
| AGAT | TAGA,GATA,TCTA,TATC,ATAG,AGAT,CTAT,ATCT |
| AGCC | GCCA,AGCC,CAGC,TGGC,CCAG,CTGG,GCTG,GGCT |
| AGCG | GCGA,GAGC,TCGC,GCTC,CGAG,AGCG,CTCG,CGCT |
| AGCT | GCTA,TAGC,CTAG,AGCT |
| AGGC | GGCA,TGCC,AGGC,CTGC,GCAG,CAGG,CCTG,GCCT |
| AGGG | GGGA,TCCC,CTCC,CCTC,GGAG,GAGG,AGGG,CCCT |
| ATAT | TATA,ATAT |
| ATCC | TCCA,TGGA,ATCC,CATC,ATGG,GATG,CCAT,GGAT |
| ATCG | TCGA,GATC,ATCG,CGAT |
| ATGC | TGCA,ATGC,CATG,GCAT |
| CCCG | GCCC,CGCC,CCGC,GGGC,CCCG,GGCG,GCGG,CGGG |
| CCGG | GGCC,CGGC,GCCG,CCGG |
| CGCG | GCGC,CGCG |

**Table S4.** Manually curated 57 STR loci. The coordinates are the starting and ending positions of STR loci in hg38. “Chr” is the chromosome name, while “Motif” is for STR.

| **Chr** | **Start pos** | **End pos** | **Motif** | **Name** |
| --- | --- | --- | --- | --- |
| chr1 | 230,701,148 | 230,701,186 | TG | d1s103 |
| chr8 | 130,064,893 | 130,064,933 | TG | d8s344 |
| chrX | 67,545,317 | 67,545,383 | CAG | ar |
| chr12 | 6,936,728 | 6,936,773 | CAG | atn1 |
| chr6 | 16,327,633 | 16,327,723 | CAG | atxn1 |
| chr12 | 111,598,950 | 111,599,019 | CAG | atxn2 |
| chr14 | 92,071,010 | 92,071,052 | CAG | atxn3 |
| chr3 | 63,912,685 | 63,912,715 | CAG | atxn7 |
| chr13 | 70,139,351 | 70,139,429 | CTG | atxn8os |
| chr19 | 13,207,858 | 13,207,897 | CAG | cacna1a |
| chr18 | 3,990,629 | 3,990,665 | ATA | d18s853 |
| chr1 | 106,421,091 | 106,421,130 | ATT | d1s1627 |
| chr20 | 55,249,398 | 55,249,440 | ATA | d20s1082 |
| chr22 | 37,140,286 | 37,140,329 | ATT | d22s1045 |
| chr8 | 42,681,445 | 42,681,472 | AAT | d8s1115 |
| chr9 | 133,160,281 | 133,160,311 | ATA | d9s2157 |
| chr19 | 45,770,204 | 45,770,264 | CTG | dmpk |
| chrX | 147,912,050 | 147,912,110 | CGG | fmr1 |
| chr9 | 69,037,286 | 69,037,304 | GAA | fxn |
| chr4 | 3,074,876 | 3,074,933 | CAG | htt |
| chr5 | 146,878,728 | 146,878,758 | CAG | ppp2r2b |
| chr6 | 170,561,906 | 170,562,017 | CAG | tbp |
| chr5 | 150,076,321 | 150,076,373 | CTAT | csf1po |
| chr10 | 129,294,243 | 129,294,295 | GGAA | d10s1248 |
| chr10 | 2,201,137 | 2,201,181 | TATC | d10s1435 |
| chr11 | 131,002,508 | 131,002,560 | TCTA | d11s4463 |
| chr12 | 12,297,021 | 12,297,093 | ATAG | d12s391 |
| chr14 | 94,842,054 | 94,842,114 | TCTA | d14s1434 |
| chr16 | 86,352,701 | 86,352,745 | GATA | d16s539 |
| chr17 | 74,684,854 | 74,684,902 | AGAT | d17s1301 |
| chr19 | 15,617,483 | 15,617,534 | ATCT | d19s253 |
| chr1 | 163,590,025 | 163,590,102 | TTCC | d1s1677 |
| chr20 | 4,525,691 | 4,525,750 | AGAT | d20s482 |
| chr20 | 39,423,110 | 39,423,187 | TTTC | d20s85 |
| chr2 | 17,310,717 | 17,310,801 | AGAT | d2s1360 |
| chr2 | 168,788,892 | 168,788,936 | AGAT | d2s1776 |
| chr2 | 68,011,946 | 68,011,994 | TCTA | d2s441 |
| chr3 | 45,540,736 | 45,540,803 | TATC | d3s1358 |
| chr3 | 172,033,173 | 172,033,209 | AGAT | d3s3053 |
| chr3 | 85,803,482 | 85,803,534 | AGAT | d3s4529 |
| chr4 | 31,302,797 | 31,302,833 | ATCT | d4s2408 |
| chr5 | 59,401,443 | 59,401,487 | CTAT | d5s2500 |
| chr5 | 123,775,552 | 123,775,599 | TCTA | d5s818 |
| chr6 | 41,709,529 | 41,709,569 | TGGA | d6s1017 |
| chr6 | 91,740,222 | 91,740,270 | CTAT | d6s1043 |
| chr6 | 112,557,949 | 112,558,016 | TAGA | d6s474 |
| chr8 | 124,894,862 | 124,894,914 | TATC | d6s502 |
| chr7 | 123,857,644 | 123,857,732 | CTTT | d7s1517 |
| chr7 | 84,160,224 | 84,160,276 | CTAT | d7s820 |
| chr8 | 106,316,691 | 106,316,774 | TCTA | d8s1132 |
| chr8 | 124,894,862 | 124,894,917 | TATC | d8s1179 |
| chr8 | 16,913,542 | 16,913,663 | ATAG | d8s639 |
| chr9 | 77,073,825 | 77,073,873 | TAGA | d9s1122 |
| chr11 | 2,171,085 | 2,171,113 | TGAA | humth01 |
| chr2 | 1,489,650 | 1,489,682 | TGAA | tpox |
| chr22 | 45,795,354 | 45,795,424 | ATTCT | atxn10 |
| chr10 | 12,751,051 | 12,751,126 | ATAAG | d10s2325 |

**Table S5.** Sequencing error statistics of the G4C2 repeat region (Table in a separate Excel file).
