# Supplementary material for: DeepRepeat: direct quantification of short tandem repeats on signal data from nanopore sequencing
Source: Genome Biol. 2022 Apr 28;23:108. doi: 10.1186/s13059-022-02670-6 (PMC9052667; doi:10.1186/s13059-022-02670-6)
Supplement: Supplementary file 4 — Additional file 4. Review history. [file 13059_2022_2670_MOESM4_ESM.docx]

**1^st^ round**

**Reviewer 1**

Summary and general comments:

Liu et al present the tool DeepRepeat to quantify the length of tandem repeats using raw nanopore signals. The tool is competitive with other existing methods and appears to be a significant improvement in speed. My major concern is that only a small number of loci are ever evaluated in any one of the results sections. Demonstration of the method genome wide with a reliable test/validation set would significantly improve the paper. The publicly available nanopore data for the T2T-CHM13 assembly with more than 150,000 large tandem repeats would allow the authors to address this concern.

Specific concerns:

1. Figure 2's legend should be improved. It is unclear what the colors are being used for and the difference between the data in the different panels, and makes it quite difficult to evaluate the authors conclusions.

2. Without more detail I am unsure if the increased error rates shown in "estimation of mismatch error or repeat regions" are due to poor alignment or actually increased error rates. Are the synthetic sequences and their references matched (representative of the same haplotypes) or do they simply share repeat counts? For me, showing simple IGV screenshots of the tandem repeat alignments in the supplement could be very convincing. Additionally a summary table of the change in error rate would be informative and help quantify the finding.

3. The use of telomeric repeats in CHM13 v1.1 as a benchmark seems potentially problematic since we expect the telomere repeat length to be mosaic in the CHM13 (and other) cell lines. Can the authors comment on whether they observed heterogeneity? Also the results are only described as highly accurate without quantification, which I think is necessary.

4. In the CHM13 evaluation section what data was used to train DeepRepeat? I think it would be good to include this while presenting the results. In fact, throughout the entire results section I think it would be important to state what training data was used for DeepRepeat with each presented result.

5. The use of only a couple repeat regions to evaluate DeepRepeat throughout the paper is concerning. I think that paper would be much more convincing if the accuracy of DeepRepeat was evaluated across the entire T2T CHM13 assembly using the CHM13 nanopore data. I count > 150,000 tandem repeats in CHM13 v1.1 with at least 50bp and of repeat sequence (and 1500 greater than 300bp) which I think could be used as an excellent test set to strengthen the paper. I see that in the methods several steps were taken to filter down to the 20 regions reported on in the paper, but I think that it is important that the method is shown to be of use on a more global dataset instead of just a highly filtered one.

6. Paneles d and e in figure 5 would be improved if a vertical line with the true copy number was included.

7. The color used for DeepRepeat in figure 5 is not consistent (both blue and red). It would be helpful if this was changed to be consistent.

8. A discussion of how imperfect tandem repeats are handled would be important since long arrays are particularly prone to having impurities, or even multiple motifs.

9. In "evaluate DeepRepeat on 9 STR loci in NA12878 with Sanger sequencing" the discussed results are only the positive results from Table 1. I think it would be important to include in the text how all these methods perform across these loci compared to Sanger since clearly there are some cases where the other methods outperform DeepRepeat.

10. "Cross-genome evaluation of DeepRepeat for repeat-nucleotide prediction" does not include an evaluation of the other methods for comparison which makes the precision and recall hard to interpret. Can a smaller comparison be made? Maybe just using chr20 for example?

**Reviewer 2**

Liu et al used a creative idea to transform a repeat-recognition problem into an image-recognition one so that deep learning could be applied. The resulting software, DeepRepeat, was designed to recognize microsatellite sequences from Nanopore raw electric signals. The authors systematically tested the performance of DeepRepeat on public and their own data, and showed that it has a unique ability to identify telomeric repeats while showing favorable metrics over alternative software on other types of simple tandem repeats (STRs). The work was presented clearly and easy to follow. I haven't tried their software (for that I need some labeled data), but it is openly accessible and well documented.

Major comments:

1. The manuscript could be benefit quite a bit from language editing. I've included some edits to the abstract (see minor , but the entire manuscript should be revised to improve clarity. Besides this, some parts of the text could be moved to supplemental sections. For example, the section describe the Oxford ONT sequencing error rate in the STR regions is higher (p1L133-p8L177 and Figure 2). Not sure Figure 3 is even needed.

2. Is DeepRepeat the only available software that can reliably detect telomeric repeat copy numbers? If so, please state that. This will have tremendous implications for aging research. What is its pros and cons compared with traditional PCR-based strategies? (similarly, compare with PCR-based repeat expansion identification in Huntington Diseases)

3. Some discussions about a generic model that recognizes all STRs instead of requiring a model for each STR would be good. Intuitively it should be possible as DeepRepeat is based on self-similarity of adjacent units.

4. Could DeepRepeat benefit from GPU when used in the training/prediction?

Minor comments:

p2L25 "on genomic regions with long expanded repeats," -> on genomic regions with long STRs

p2L26 "pathogenic repeat counts" -> pathogenic repeat copies

p2L27 "or other complex genomic regions, where flanking sequences" -> or other low-complexity genomic regions, where flanking sequences

p2L31-32 "by modeling multiple reads with a Gaussian mixture model. As a unique application of DeepRepeat, " -> by modeling multiple reads with a Gaussian mixture model. To validate the accuracy of DeepRepeat,

p2L38 "long STR loci in complex genomic regions" -> long STR loci in low-complexity genomic regions

p4L77-78 "they are becoming much 77 higher in repetitive regions" -> is this statement true for both PacBio and Oxford ONT sequencing? If so, supporting evidence needs to be provided

p8L178 "Detection of long repeat regions in CHM13 " -> Detection of long STRs in CHM13

p9L180 "studied haploid (largely homozygous, with minor exceptions in specific regions) genome " -> well, a largely homozygous diploid is NOT a haploid, and genome by definition is always haploid

p10L212 " we generated high coverage (~4000X) " -> please justify why 4000x is needed or not

**Authors’ response**

Reviewer #1

Summary and general comments:

Liu et al present the tool DeepRepeat to quantify the length of tandem repeats using raw nanopore signals. The tool is competitive with other existing methods and appears to be a significant improvement in speed. My major concern is that only a small number of loci are ever evaluated in any one of the results sections. Demonstration of the method genome wide with a reliable test/validation set would significantly improve the paper. The publicly available nanopore data for the T2T-CHM13 assembly with more than 150,000 large tandem repeats would allow the authors to address this concern.

Authors’ Response: Thanks for your time and valuable comments, and we completely agree that additional evaluation using the high-quality T2T assembly would greatly improve the paper. Our response to your major concern can be found in #5 below.

Specific concerns:

1. Figure 2's legend should be improved. It is unclear what the colors are being used for and the difference between the data in the different panels, and makes it quite difficult to evaluate the authors conclusions.

Authors’ Response: Thanks for this comment. We improved both figure legend and the figure. Originally, colors were used for longer and shorter repeat copies in different panels, and different panels had different repeat copies. To make it clearer, we now remove color information, and add the information of repeat copies in the figure. Please refer to Page 23 for detail.

2. Without more detail I am unsure if the increased error rates shown in "estimation of mismatch error or repeat regions" are due to poor alignment or actually increased error rates. Are the synthetic sequences and their references matched (representative of the same haplotypes) or do they simply share repeat counts? For me, showing simple IGV screenshots of the tandem repeat alignments in the supplement could be very convincing. Additionally a summary table of the change in error rate would be informative and help quantify the finding.

Authors’ Response: Thanks for this comment. We added IGV screenshots Supplementary Figure 2 and Supplementary Figure 3. We also provided the error rate details for the 8 vs 76 repeat copies, and the 15 vs 800 repeat copies basecalled by both Guppy 3 and Guppy 5 (Supplementary Table 5, as a separate Excel file).

3. The use of telomeric repeats in CHM13 v1.1 as a benchmark seems potentially problematic since we expect the telomere repeat length to be mosaic in the CHM13 (and other) cell lines. Can the authors comment on whether they observed heterogeneity? Also the results are only described as highly accurate without quantification, which I think is necessary.

Authors’ Response: Thanks for this insightful comment. In the original manuscript we only analyzed one telomere region. In the revised manuscript, we added the quantification of all telomere regions of CHM13 (Figure 3a, Page 24) and compared the results with the CHM13 assembly. In general, DeepRepeat’s quantification is consistent with the CHM13 v1.1 assembly (Pearson correlation = 0.83), but with some variation, and we discussed the possibility of mosaicism of telomere length (Page 6, lines 187-203). We also observed that the variation of different reads within the same telomere region is higher than regular repeat regions (standard deviation of repeat count is about 100), also suggesting the possibility of mosaicism. Nevertheless, since high-quality T2T v1.1 assembly can be generated by the T2T consortium, the extent of mosaicism does not appear to have much influence on assembly. Also, for a given telomere, the repeat counts are centered around one peak and we did not observe multimodal pattens.

4. In the CHM13 evaluation section what data was used to train DeepRepeat? I think it would be good to include this while presenting the results. In fact, throughout the entire results section I think it would be important to state what training data was used for DeepRepeat with each presented result.

Authors’ Response: Thanks for this comment. We added the description of the training data in the first paragraph of each evaluation section (yellow highlighted throughout the manuscript). For general repeat analysis, DeepRepeat was trained on two wholegenome Nanopore data sets (NA12878 and HX1). For detection of the CAG repeats from Huntington’s disease (HD) samples, DeepRepeat was trained on a large HD data set generated in house. For detection of the GGGGCC repeats, DeepRepeat was trained on the samples with fewer number of repeats and was tested on the samples with the larger number of repeats. For end users, DeepRepeat will automatically chose the model based on the input.

5. The use of only a couple repeat regions to evaluate DeepRepeat throughout the paper is concerning. I think that paper would be much more convincing if the accuracy of DeepRepeat was evaluated across the entire T2T CHM13 assembly using the CHM13 nanopore data. I count > 150,000 tandem repeats in CHM13 v1.1 with at least 50bp and of repeat sequence (and 1500 greater than 300bp) which I think could be used as an excellent test set to strengthen the paper. I see that in the methods several steps were taken to filter down to the 20 regions reported on in the paper, but I think that it is important that the method is shown to be of use on a more global dataset instead of just a highly filtered one.

Authors’ Response: Thanks for this comment and we completely agree that it is much more convincing to evaluate a method including DeepRepeat on a larger dataset. The previous version of the manuscript used 20 regions that were manually examined. In the revised manuscript, we benchmarked DeepRepeat, RepeatHMM and STRique on 439 STR regions on the CHM13 genome. These are all STR regions that are > 200 bp and not within a 500 bp flanking region of another STR. We removed adjacent STRs because many of the adjacent STRs have similar sequences and it’s hard to tell if they need to be merged or not and therefore it’s hard to infer the gold standard repeat count. The length of the 439 STR regions range from 200 bp to 2374 bp. The evaluation results are shown in Figure 3 (Page 24) and the results are described in Page 6 (lines 205-215). In summary, by addressing this comment, we have substantially improved this section.

6. Paneles d and e in figure 5 would be improved if a vertical line with the true copy number was included.

Authors’ Response: Thanks for this comment. We add down arrows in magenta to show the true copy numbers, and the true copy numbers are described in the figure legend (new Figure 4, see Page 25).

7. The color used for DeepRepeat in figure 5 is not consistent (both blue and red). It would be helpful if this was changed to be consistent.

Authors’ Response: Thanks for this comment. The original Figure 5 is Figure 4 in the revised manuscript. We have modified the figure and the colors of different methods are now consistent in Figure 4(a), (b), (d) and (e). Please refer to Page 25.

8. A discussion of how imperfect tandem repeats are handled would be important since long arrays are particularly prone to having impurities, or even multiple motifs.

Authors’ Response: Thanks for this comment. We have now discussed the two types of imperfect tandem repeats in Page 14 (lines 586-592). First, within DeepRepet, STR motifs of repeat units are usually considered as a group rather than individually, so an imperfect tandem repeat may still have repeat units in the same group. Second, the pixel generation routine can accommodate some levels of impurities: in an ideal case, without impurities, the color image of three consecutive repeat units would only have R white dots where the three channels all have 255 at the R positions and 0 at all other positions in repeat images; if there is slight disruption, then noises are introduced in the image as if there are gray dots in a white line, but the algorithm can still recognize the pattern as a white line.

9. In "evaluate DeepRepeat on 9 STR loci in NA12878 with Sanger sequencing" the discussed results are only the positive results from Table 1. I think it would be important to include in the text how all these methods perform across these loci compared to Sanger since clearly there are some cases where the other methods outperform DeepRepeat.

Authors’ Response: Thanks for this comment. Since the 9 STR loci do not have long STR regions, other methods (including Sanger, HipSTR, Tandem-genotyper, RepeatHMM) were able to achieve good performance. Per your suggestions, we add discussion of other methods in Page 9 (lines 325-327).

10. "Cross-genome evaluation of DeepRepeat for repeat-nucleotide prediction" does not include an evaluation of the other methods for comparison which makes the precision and recall hard to interpret. Can a smaller comparison be made? Maybe just using chr20 for example?

Authors’ Response: Thanks for this comment. Whenever feasible, we always compare our method against other competing methods. However, this particular evaluation is not about quantifying repeats; instead, it is for the prediction of whether a single base is in a repeat (using the specific signal patterns of repeat regions). This is a unique feature of DeepRepeat and may find some utility in some applications. Other methods do not make this type of prediction, so we can only perform the genome-wide comparison on STRs with other methods as previously mentioned. We added the clarification in Page 9, lines 353-355.

Reviewer #2

Liu et al used a creative idea to transform a repeat-recognition problem into an imagerecognition one so that deep learning could be applied. The resulting software, DeepRepeat, was designed to recognize microsatellite sequences from Nanopore raw electric signals. The authors systematically tested the performance of DeepRepeat on public and their own data, and showed that it has a unique ability to identify telomeric repeats while showing favorable metrics over alternative software on other types of simple tandem repeats (STRs). The work was presented clearly and easy to follow. I haven't tried their software (for that I need some labeled data), but it is openly accessible and well documented.

Authors’ Response: Thank you very much for your nice comments.

Major comments:

1. The manuscript could be benefit quite a bit from language editing. I've included some edits to the abstract (see minor , but the entire manuscript should be revised to improve clarity. Besides this, some parts of the text could be moved to supplemental sections. For example, the section describe the Oxford ONT sequencing error rate in the STR regions is higher (p1L133-p8L177 and Figure 2). Not sure Figure 3 is even needed.

Authors’ Response: Thanks for this comment. We have gone through the manuscript and improve the clarity, including the section describing the Oxford ONT sequencing error rate. Additionally, we have removed Figure 3 as suggested (since we still feel that this is an illustrative figure for readers to gain intuition how the method works, we now include it in Supplementary Materials).

2. Is DeepRepeat the only available software that can reliably detect telomeric repeat copy numbers? If so, please state that. This will have tremendous implications for aging research. What is its pros and cons compared with traditional PCR-based strategies? (similarly, compare with PCR-based repeat expansion identification in Huntington Diseases)

Authors’ Response: Thanks for this comment. Based on our current evaluation, DeepRepeat is the only method to reliably estimate repeat copies in telomere since other repeat detection methods need both flanking regions (see Page 6 for descriptions and some discussions). Assembly-based methods (e.g. CHM13 assembly) can obviously also give an estimation of repeat counts in assembly, but does not provide repeat count details of each read. As the telomeric repeats might be mosaic, DeepRepeat gives more information about the distribution of the repeat length. qPCR-based methods cannot distinguish telomeric repeats from different chromosomes and thus the quantification is an average repeat count of all telomeres. DeepRepeat can detect the repeat count of each telomere, based on the mapping of nanopore reads. The cons are that Nanopore sequencing has much higher cost than traditional qPCR methods, it is difficult to perform amplicon-based or capture-based Nanopore sequencing for telomeric regions, and that it requires more computational resources for data analysis on whole-genome data. See Page 11 Paragraph 2 for additional discussions.

The traditional method for Huntington’s disease repeat quantification is PCR-based fragment analysis. Long-read sequencing has the advantage of high throughput. In our internal experiments, we can multiplex and sequence 500 HD samples per MinION flow cell, by using barcoded primers and high-fidelity long-range PCR. The PCR product can be several kb. In addition, we can call haplotyped genetic variants from long reads, including repeat expansions and SNPs. This has strong clinical implications since understanding the haplotype structure of a repeat region allows design of allele-specific therapeutic approaches. Traditional methods such as Sanger sequencing cannot provide haplotype information because they can only assay a 500- 1000bp region, despite higher accuracy in SNP and indel detection.

3. Some discussions about a generic model that recognizes all STRs instead of requiring a model for each STR would be good. Intuitively it should be possible as DeepRepeat is based on self-similarity of adjacent units.

Authors’ Response: Thanks for this comment. We agree with the reviewer that it is possible to have generic model for all STRs if a suitable training data is available. In this study, we provide different well-trained repeat models according to the length of the repeat motifs and the motif groups. Currently, DeepRepeat will automatically chooses the model according to the input repeat motif. In the future, a generic DeepRepeat model can be provided for all possible STR motifs, since DeepRepeat is based on self-similarity of neighborhood signals. However, the suitable training datasets (possibly synthetic sequences) are still needed. Right now, we generated training data from NA12878 and HX1, but the occurrence instances of different motifs are highly unbalanced, and longer STR motifs have much less occurrences. Thus, it is not easy to have a balanced dataset to train a generic model, while motif-specific trained models can provide better estimation. To further address this comment, we add this discussion in Page 11 per your suggestions (lines 445-453).

4. Could DeepRepeat benefit from GPU when used in the training/prediction?

Authors’ Response: Thanks for this comment. We trained DeepRepeat on GPU and tested it on both CPU and GPU. Using GPU can reduce the running time. When users want to train their own DeepRepeat model, we recommend using GPU to accelerate the training process.

Minor comments:

p2L25 "on genomic regions with long expanded repeats," -> on genomic regions with long STRs

p2L26 "pathogenic repeat counts" -> pathogenic repeat copies

p2L27 "or other complex genomic regions, where flanking sequences" -> or other lowcomplexity genomic regions, where flanking sequences

p2L31-32 "by modeling multiple reads with a Gaussian mixture model. As a unique application of DeepRepeat, " -> by modeling multiple reads with a Gaussian mixture model. To validate the accuracy of DeepRepeat,

p2L38 "long STR loci in complex genomic regions" -> long STR loci in low-complexity genomic regions

Authors’ Response: Thanks very much for correcting the sentences. We have corrected all the issues and also checked the whole manuscript to improve the clarifications.

p4L77-78 "they are becoming much 77 higher in repetitive regions" -> is this statement true for both PacBio and Oxford ONT sequencing? If so, supporting evidence needs to be provided

Authors’ Response: Thanks for this comment. As DeepRepeat works on signal data, we evaluated Nanopore sequencing and provided supporting evidence in the manuscript, but we did not check PacBio sequencing data. We thus improve the clarification of this statement in the manuscript. Please refer to Page 5 (line 174). p8L178 "Detection of long repeat regions in CHM13 " -> Detection of long STRs in CHM13

Authors’ Response: Thanks for this comment. We have made the correction.

p9L180 "studied haploid (largely homozygous, with minor exceptions in specific regions) genome " -> well, a largely homozygous diploid is NOT a haploid, and genome by definition is always haploid

Authors’ Response: Thanks for this comment and we have re-worded this statement. The CHM13 cell is sometime referred to as “effectively haploid” (hence it was chosen for genome assembly in the T2T consortium), but in fact it is a largely homozygous diploid as the reviewer stated, including a large deletion. We state “The CHM13 cell line has near-complete homozygosity, with a few exceptions in specific regions”

(Please refer to Page 6, lines 180-181).

p10L212 " we generated high coverage (~4000X) " -> please justify why 4000x is needed or not

Authors’ Response: Thanks for this comment. 4000X is a lot more than needed in practice. We clarified this in Page 7 (lines 244-246). In this study, we generated this high coverage data because it is a targeted sequencing and the MinION flow cell has more capacity than needed. We envision that this approach can be used for Flongle flow cells (a smaller flow cell) for low-cost low-throughput Nanopore sequencing in the future.

**2^nd^ round**

**Reviewer 1**

The authors have satisfactorily addressed my concerns and I recommend the manuscript for publication. I find the new Figure 3 particularly compelling as well as the associated text in the results section.
